# Supplementary material for: Viruses and vectors tied to honey bee colony losses
Source: PLoS Pathog. 2026 Feb 23;22(2):e1013939. doi: 10.1371/journal.ppat.1013939 (PMC12948310; doi:10.1371/journal.ppat.1013939)
Supplement: S1 Appendix — Table A. Primer names and associated sequences for qPCR detection and relative quantification of common honey bee pathogens. Table B. Primer names and associated sequences for qPCR detection and absolute quantification of four pathogens in experimental inoculum. Table C. Dilution factor of four independent inoculum (representing portion of pupa) and copy number per ul of inoculum. Table D: B-Actin and viruses screened in inoculums. Table E: Prevalence of pathogens found in asymptomatic and symptomatic bees. Table F: Inoculums and survivorship of adult bees. Fig A: Pathogen detections by colony condition. A) Strong, B) Medium, C) Weak. Fig B: Amplification of DWV-B and ABPV targets in individual, injected pupae at time zero, 36 and 60 hours post injection. A subset of pupae were sacrificed at each time point, and used as a representative for viral infection for that specific timepoint. Fig C. A typical ‘dwindled’ colony: honey stores largely intact, queen present, a patch of brood, and adult bee abundance lacking (Photo Lamas). Fig D. A typically strong colony, showing coverage across all internal frames, and dense coverage of adult bees on hive surfaces. (Photo Lamas). Fig E: A colony which continued to dwindle after uniting with another colony. (Photo Lamas). Fig F: A typically “dwindled” unite is picture below. This colony lost approximately 6 frames of adult bee coverage between January 21st, 2025 when it was inspected by the beekeeper, and January 28th, 2025 when inspected by our research team. (Photo Lamas). Fig G: Remains of a perished colony includes a mass of recently dead bees and pallet board debris. (Photo Lamas). Fig H: An up-close picture of pallet board debris, featuring numerous Varroa. (Photo Lamas). Fig I: Immobilized adult worker bees, having recently egressed from their colony. (Photo Lamas) (DOCX) [file ppat.1013939.s001.docx]

**Supplementary Tables and Figures**

| **Table A.** Primer names and associated sequences for qPCR detection and relative quantification of common honey bee pathogens. | | | |
| --- | --- | --- | --- |
| **Target** | **Primer** | **Sequence** | **Source** |
| Actin | AMActin.F | TTGTATGCCAACACTGTCCTTT | 1 |
| Actin | AMActin.R | TGGCGCGATGATCTTAATTT | 1 |
| Deformed wing virus A | DWV.F | GAGATTGAAGCGCATGAACA | 1 |
| Deformed wing virus A | DWV.R | TGAATTCAGTGTCGCCCATA | 1 |
| Deformed wing virus B | VDV1.F | GCCCTGTTCAAGAACATG | 2 |
| Deformed Wing virus B | VDV1.R | CTTTTCTAATTCAACTTCACC | 2 |
| *Nosema ceranae* | qNC40sRP.F | AGAAACTACAACAGCATCACTGGGA | 3 |
| *Nosema ceranae* | qNC40sRP.R | AGTGAATATTCCAATTCCCAACGACTT | 3 |
| Acute bee paralysis virus | ABPV.F | ACCGACAAAGGGTATGATGC | 1 |
| Acute bee paralysis virus | ABPV.R | CTTGAGTTTGCGGTGTTCCT | 1 |
| Chronic bee paralysis virus | CBPV.F | CAAAATCAACGAGCCAATCA | 1 |
| Chronic bee paralysis virus | CBPV.R | AGTGTGAGGATCACCGGAAC | 1 |
| Sacbrood virus | SBV.F | GGGTCGAGTGGTACTGGAAA | 1 |
| Sacbrood virus | SBV.R | ACACAACACTCGTGGGTGAC | 1 |
| Israeli acute paralysis virus | IAPV.F1a | GCGGAGAATATAAGGCTCAG | 1 |
| Israeli acute paralysis virus | IAPV.R1 | CTTGCAAGATAAGAAAGGGGG | 1 |
| Kashmir bee virus | KBV.F | TGAACGTCGACCTATTGAAAAA | 1 |
| Kashmir bee virus | KBV.R | TCGATTTTCCATCAAATGAGC | 1 |
| *Lotmaria passim* | Trypan1.F | CTGAGCTCGCCTTAGGACAC | 1 |
| *Lotmaria passim* | Trypan1.R | GTGCAGTTCCGGAGTCTTGT | 1 |
| *Acarapis woodi* | AcwdCO1.F | TCAATTTCAGCCTTTTATTCAAGA | 4 |
| *Acarapis woodi* | AcwdCO1.R | AAAACATAATGAAAATGAGCTACAACA | 4 |
| Lake Sinai virus | LSVrsc.F | GTCATCCCAAGAGAACCACTYAC | 5,6 |
| Lake Sinai virus | LSVrsc.R | CRCACYGACATGAAGAAATGAGGTC | 5,6 |
| Am Filamentous virus | AMFV1.F | TTATTAACACCGCAGGCTTC | 9 |
| Am Filamentous virus | AMFV1.R | CATGGTGGCCAAGTCTTGCT | 9 |
| Black queen cell virus | BQCV.F | TTTAGAGCGAATTCGGAAACA | 10 |
| Black queen cell virus | BQCV.R | GGCGTACCGATAAAGATGGA | 10 |
| P. larvae | AFB.F | CTTGTGTTTCTTTCGGGAGACGCCA | 11 |
| P. larvae | AFB.R | TCTTAGAGTGCCCACCTCTGCG | 11 |
| M. plutonius | ABC.EFB.F | TAGCAGCCCTTGTCATCGTC | 12 |
| M. plutonius | ABC.EFB.R | CCAATGGCTTGAGCTGCTTC | 12 |
| Acarapis woodi | AcwdCO1.F | TCAATTTCAGCCTTTTATTCAAGA | 13 |
| Acarapis woodi | AcwdCO1.R | AAAACATAATGAAAATGAGCTACAACA | 13 |

| **Table B.** Additional primer names and associated sequences for qPCR detection and absolute quantification of four pathogens in experimental inocula. | | |  |
| --- | --- | --- | --- |
| **Target** | **Primer** | **Sequence** |  |
| Deformed wing virus A | DWV-F8668 | TTCATTAAAGCCACCTGGAACATC | 7 |
| Deformed wing virus A | DWV-B8757 | TGGCGCGATGATCTTAATTT | 7 |
| Deformed wing virus B (VDV-1) | qVDV1-CP-F | CTGTAGTTAAGCGGTTATTAGAA | 8 |
| Deformed wing virus B (VDV-1) | qVDV1-CP-R | GGTGCTTCTGGAATAGCGGAA | 8 |
| Black queen cell virus | BQCV-qF7893 | AGTGGCGGAGATGTATGC | 7 |
| Black queen cell virus | BQCV-qB8150 | GGAGGTGAAGTGGCTATATC | 7 |
| Acute bee paralysis virus | ABPV-F6548 | TCATACCTGCCGATCAAG | 7 |
| Acute bee paralysis virus | KIABPV-B6707 | CTGAATAATACTGTGCGTATC | 7 |

**Table B:** Primer sequences used for quantification of viral inoculum. Primer efficiencies were as follows” **DWV-A**: y = -3.428x + 37.911, **DWV-B:** y = -3.3881x + 37.28, **ABPV:** y = -3.3064x + 38.748

**References, Tables A and B**

1. Cornman, R. S., D. R. Tarpy, Y. Chen, et al. Pathogen webs in collapsing honey bee colonies. *PLoS ONE* 7, no. 8 (2012).
2. Ryabov, Eugene V, A.K. Childers, D. Lopez, et al. Dynamic evolution in the key honey bee pathogen deformed wing virus: novel insights into virulence and competition using reverse genetics. *PLoS Biology* 17, no. 10 (2019): e3000502.
3. Schwarz, R. S., N. A. Moran, and J. D. Evans. Early gut colonizers shape parasite susceptibility and microbiota composition in honey bee workers. *Proceedings of the National Academy of Sciences of the United States of America* 113, no. 33 (2016): 9345–50. <https://doi.org/10.1073/pnas.1606631113>.
4. Evans, J. D., J. S. Pettis, and I. B. Smith. A diagnostic genetic test for the honey bee tracheal mite, *Acarapis woodi*. *Journal of Apicultural Research* 46, no. 3 (2007): 195–97.
5. Iwanowicz, Deborah D, Judy Y Wu-Smart, Tugce Olgun, et al. “An updated genetic marker for detection of Lake Sinai virus and metagenetic applications.” *PeerJ* 8 (2020): e9424.
6. Stamets, Paul E, Nicholas L Naeger, Jay D Evans, et al. “Extracts of polypore mushroom mycelia reduce viruses in honey bees.” *Scientific Reports* 8, no. 1 (2018): 1–6.
7. Locke, B., E. Forsgren, and J. R. De Miranda. Increased tolerance and resistance to virus infections: a possible factor in the survival of varroa destructor-resistant honey bees (*Apis mellifera)*. *PLoS ONE* 9, no. 6 (2014).
8. Ryabov EV, Wood GR, Fannon JM, Moore JD, Bull JC, Chandler D, et al. A virulent strain of deformed wing virus (DWV) of honeybees (*Apis mellifera*) prevails after *Varroa destructor*-mediated, or in vitro, transmission*. PLoS Path.* (2014) 10:e1004230. doi: 10.1371/journal.ppat.1004230.
9. Wintermantel, D., Locke, B., Andersson, G. K., Semberg, E., Forsgren, E., Osterman, J., ... & de Miranda, J. R. (2018). Field-level clothianidin exposure affects bumblebees but generally not their pathogens. Nature Communications, 9(1), 5446.
10. Traynor KS, Rennich K, Forsgren E, Rose R, Pettis J, Kunkel G, et al. Multiyear survey targeting disease incidence in US honey bees. Apidologie. 2016;47: 325–347. doi:10.1007/s13592-016-0431-0
11. D. C. De Graaf, A. M. Alippi, K. Antúnez, K. A. Aronstein, G. Budge, D. De Koker, et al. *Journal of Apicultural Research* 2013; 52: 1-28
12. Evans, J. D., J. S. Pettis, and I. B. Smith (2007) A diagnostic genetic test for the honey bee tracheal mite, *Acarapis woodi*. *Journal of Apicultural Research* 46:195-197

| **Table C.** Dilution factor of four independent inoculum (representing portion of pupa) and copy number per ul of inoculum | | | | | | | |
| --- | --- | --- | --- | --- | --- | --- | --- |
| **Inoculum** | **Virus** | **10^−4^** | **10^−5^** | **10^−6^** | **10^−7^** | **10^−8^** | **10^−9^** |
| Inoculum A | DWV-B | 843,000 | 84,300 | 8,430 | Na | Na | Na |
| Inoculum B | DWV-B | 227,664 | 22,766 | 2,276 | Na | Na | Na |
| Inoculum C | DWV-A | 70,900 | 7,090 | 709 | 70.9 | 7.09 | < 1 |
|  | DWV-B | 893,000 | 89,300 | 8,930 | 893 | 89.3 | 8.9 |
|  | ABPV | 3,520,000 | 352,000 | 35,200 | 3,520 | 352 | 35.2 |
| Inoculum D | NaN | NaN | NaN | NaN | NaN | NaN | NaN |

**Table C:** Bee equivalents and copy number (per ul of inoculum) for each viral target detected per inoculum.

| **Table D. Ct values of B Actin and pathogen targets in inoculum prep** | | |
| --- | --- | --- |
| **Inoculum** | **Virus** | **Ct** |
| Inoculum A | B Actin | 31.33 |
|  | ABPV |  |
|  | AmFV | 42.78933 |
|  | BQCV |  |
|  | CBPV | - |
|  | DWV-A | - |
|  | DWV-B | 27.00 |
|  | IAPV | - |
|  | KBV | - |
|  | LSV | - |
|  | SBV |  |
| Inoculum B | B Actin | 28.91 |
|  | ABPV | 38.86 |
|  | AmFV | 42.05 |
|  | BQCV | **-** |
|  | CBPV | **-** |
|  | DWV-A | - |
|  | DWV-B | 28.56 |
|  | IAPV | - |
|  | KBV | - |
|  | LSV | - |
|  | SBV |  |
| Inoculum C | B Actin | 26.69 |
|  | ABPV | 27.08 |
|  | AmFV | 44.10 |
|  | BQCV |  |
|  | CBPV | - |
|  | DWV-A | 31.56 |
|  | DWV-B | 26.53 |
|  | IAPV | - |
|  | KBV | - |
|  | LSV | - |
|  | SBV | - |
| Inoculum D | B Actin | 31.37 |
|  | ABPV | - |
|  | AmFV | 36.19 |
|  | BQCV | - |
|  | CBPV | - |
|  | DWV-A | - |
|  | DWV-B | - |
|  | IAPV | - |
|  | KBV | - |
|  | LSV | - |
|  | SBV | - |

**Table D:** B-Actin and viruses screened in inoculums.

| **Table E. Prevalence of pathogens in individually collected bees** | | | |
| --- | --- | --- | --- |
| **Bee Health** | **Pathogen** | **Positive Detections** | **Prevalence** |
| **Asymptomatic (N = 28)** | ABPV | 21 | 75% |
|  | AmFV | 3 | 10.7% |
|  | BQCV | 21 | 75% |
|  | DWV-A | 8 | 28.6% |
|  | LSV | 1 | 3.6% |
|  | *N. ceranae* | 19 | 67.9% |
|  | SBV | 4 | 14.3% |
|  | *L. passim* | 1 | 3.6% |
| **Morbid**  **(N = 38)** | ABPV | 34 | 89.5% |
|  | AmFV | 9 | 26.4% |
|  | BQCV | 29 | 76.3% |
|  | CBPV | 9 | 23.7% |
|  | DWV-A | 30 | 78.9% |
|  | DWV-B | 38 | 100% |
|  | LSV | 16 | 42.1% |
|  | *N. ceranae* | 32 | 84.2% |
|  | SBV | 11 | 28.9% |
|  | *L. passim* | 8 | 21.1% |

**Table E:** Prevalence of identified pathogens found in asymptomatic and symptomatic bees.

| **Table F. Inoculums and survivorship of adult bees** | | | |
| --- | --- | --- | --- |
| Inoculum | Concentration (Bee Equivalent) | Kaplan-Meier survival analysis (p – value) | Number (Percent Perished) |
| InoculumA | 10^-4^ | *p = 0.0025* | 8 **(50%)** |
| InoculumA | 10^-5^ | *p < 0.0001* | 13 **(81.2%)** |
| InoculumA | 10^-6^ | *p < 0.0001* | 13 **(81.2%)** |
| InoculumB | 10^-4^ | *p < 0.0001* | 14 **(87.5%)** |
| InoculumB | 10^-5^ | *p < 0.0001* | 13 **(81.2%)** |
| InoculumB | 10^-6^ | *p = 0.0009* | 9 **(56.2%)** |
| InoculumC | 10^-4^ | *p < 0.0001* | 16 **(100%)** |
| InoculumC | 10^-5^ | *p < 0.0001* | 16 **(100%)** |
| InoculumC | 10^-6^ | *p < 0.0001* | 16 **(100%)** |
| InoculumC | 10^-7^ | *p < 0.0001* | 16 **(100%)** |
| InoculumC | 10^-8^ | *p = 0.005* | 7 **(43.8%)** |
| InoculumC | 10^-9^ | *p = 0.1673* | 2 **(12.5%)** |
| InoculumD | 10^-4^ | *p = 0.0009* | 1 **(6.25%)** |
| InoculumD | 10^-5^ | *p = 1.00* | 0 **(0 %)** |
| InoculumD | 10^-6^ | *p = 1.00* | 0 **(0 %)** |

**Fig A. Pathogen detections of pooled bees by colony condition**

**
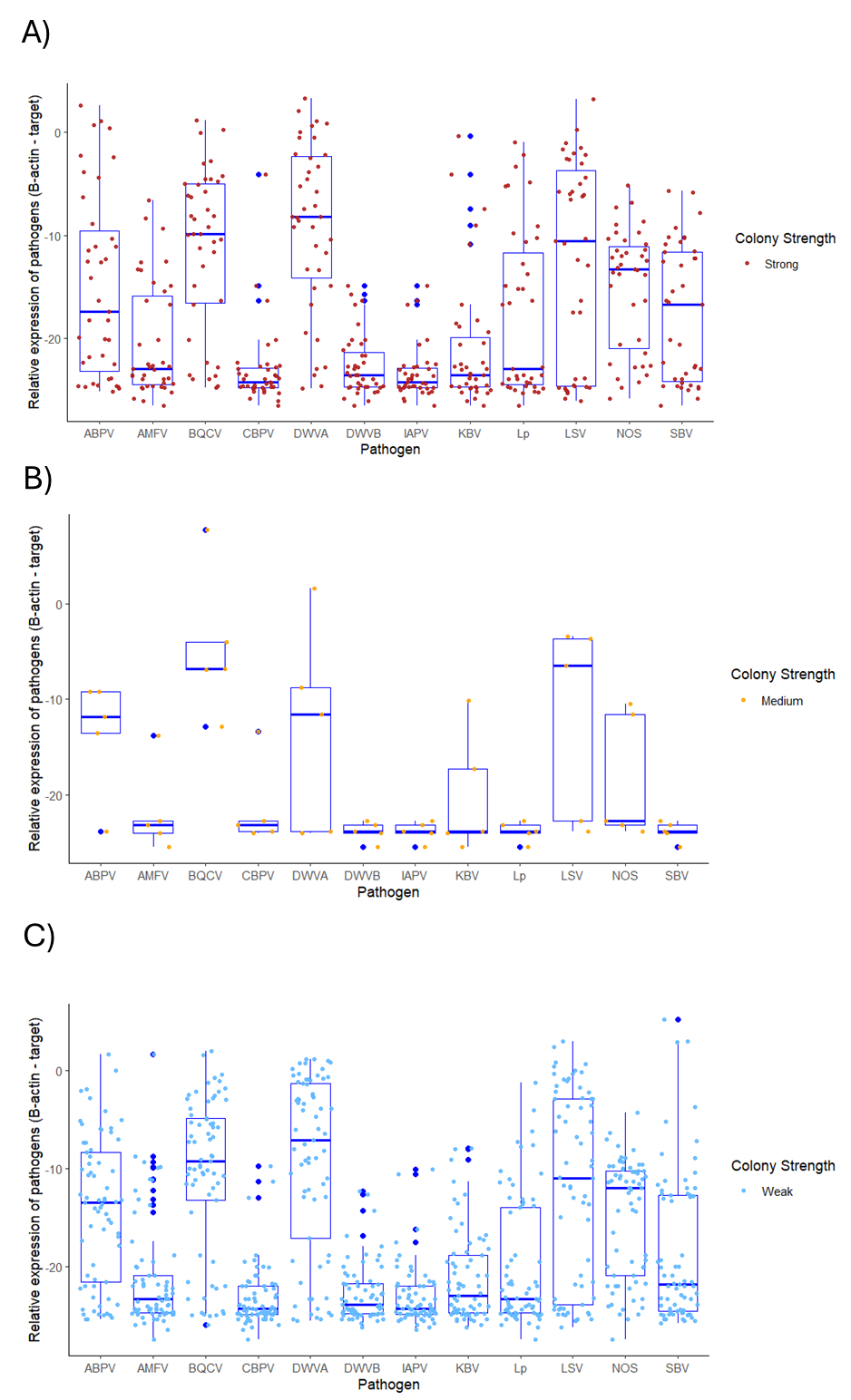
**

**Fig A:** Pathogen detections by colony condition. **A)** Strong, **B)** Medium, **C)** Weak.

**Figure B**

**
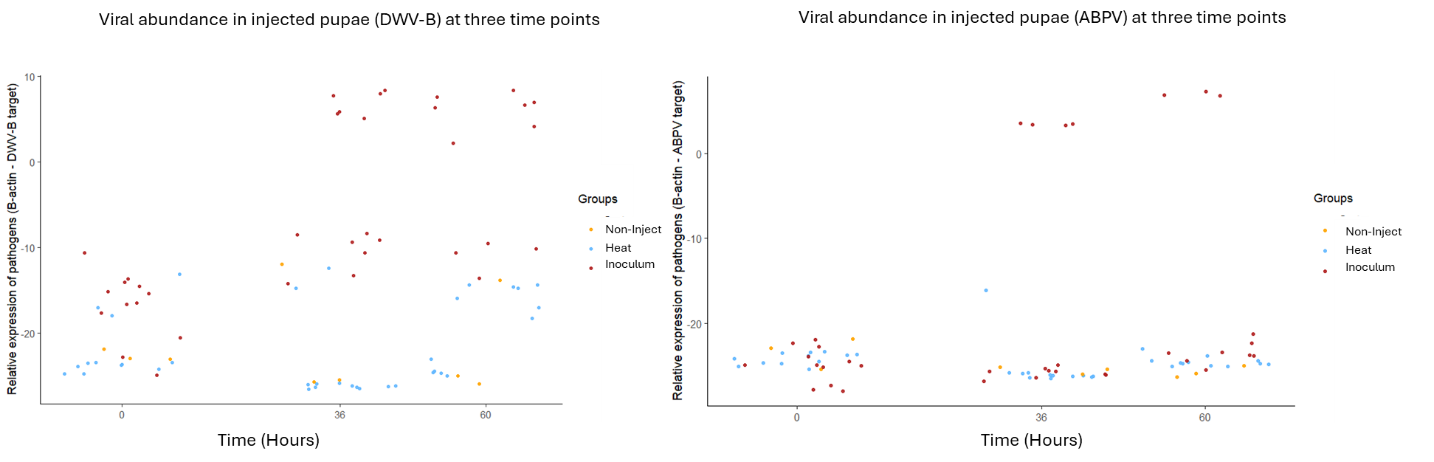
**

**Fig B.** Amplification of DWV-B and ABPV targets in individual, injected pupae at time zero, 36 and 60 hours post injection. A subset of pupae were sacrificed at each time point, and used as a representative for viral infection for that specific timepoint.

**Colony Descriptions**

Colonies generally lost the abundance of adult bees normally present within their winter cluster. The measure of these bees is not fully known due to the cross sectional design of this study. We relied upon beekeeper interviews and records to derive qualitative descriptions of loss. Below we highlight colony descriptions which convey the severity of loss while accurately portraying field observations made between January 24^th^ and February 1^st^, 2025.

1. **Typical dwindled colony**

Colonies which dwindled lost the abundance of adult bees from their winter clusters. Operation 1 colonies dwindled over the course of months. Operation 1 moved their bees from summer locations in late October to their wintering yards in California; only moving colonies in surplus of 10 frames of adult bee coverage to the state. These colonies typically were small by the January survey, with a low density of adult bees covering comb surfaces. In some instances, brood was not sufficiently covered by adult bees. Brood was occasionally symptomatic, though it was unclear if morbidities were from an underlying pathology, lack of attendance from insufficient worker bees, or a combination of both. Operation 1 fed sucrose to colonies prior to sampling.

**
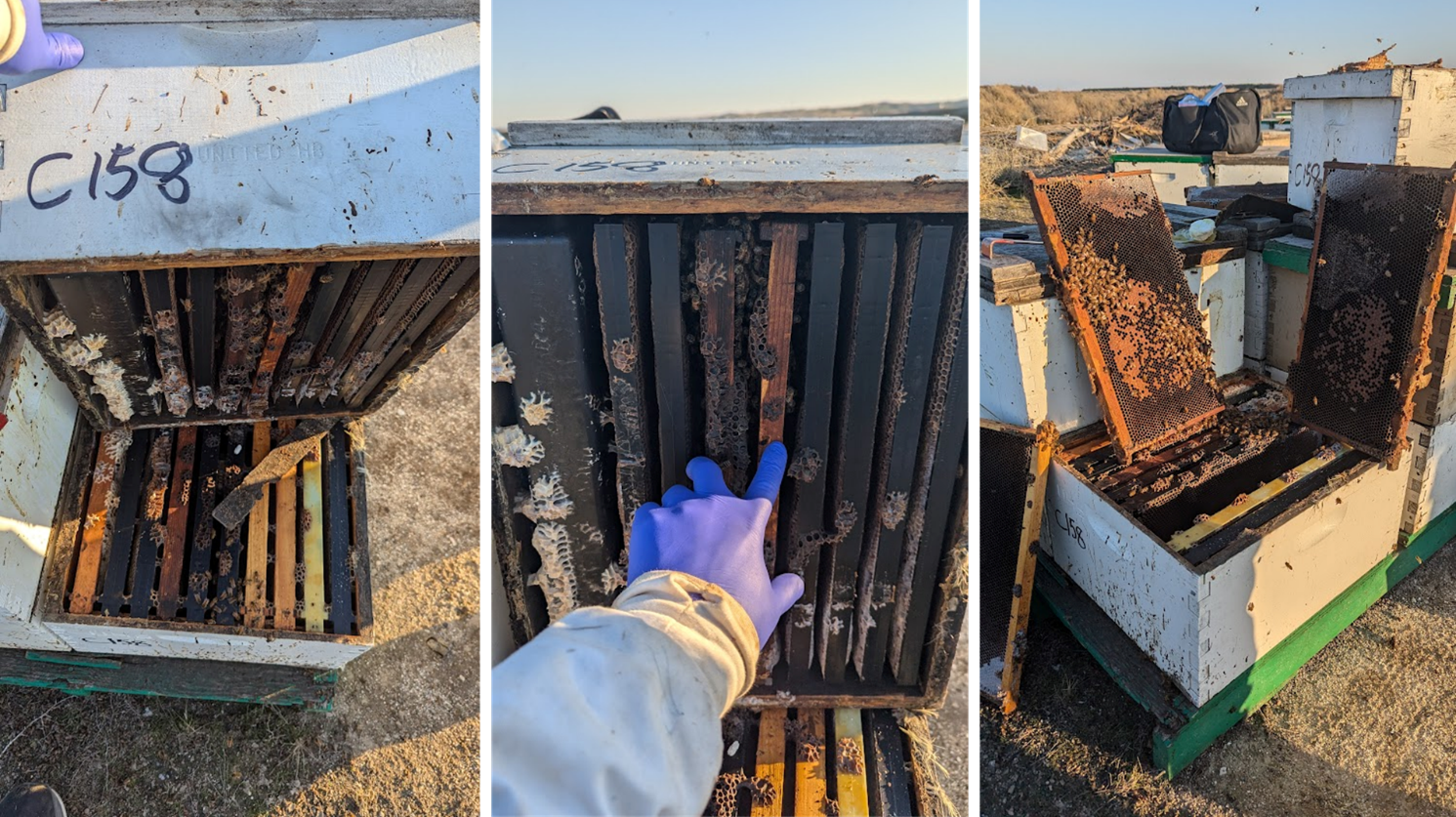
**

**Fig C.** A typical weak, ‘dwindled’ colony: honey stores largely intact, queen present, a patch of brood, and adult bee abundance lacking.

1. **Typical strong colony**

Colonies identified as strong typically had robust adult bee populations, completely covering the entire surface of the interior frames of the colony. Although quantitative estimates of adult bees were not possible, a qualitative description of dense bee coverage would accurately describe colonies rated as strong. We used strong as a qualifier during field assessments. This description is interchangeable with “unaffected” in that the colonies appeared to not suffer a rapid loss of adult bees. Brood was not a good indicator of colony strength as colonies recently removed from indoor winter storage had little, and queens in field colonies may have had intermittent laying, likely from the ebbs and flows of winter weather.

**
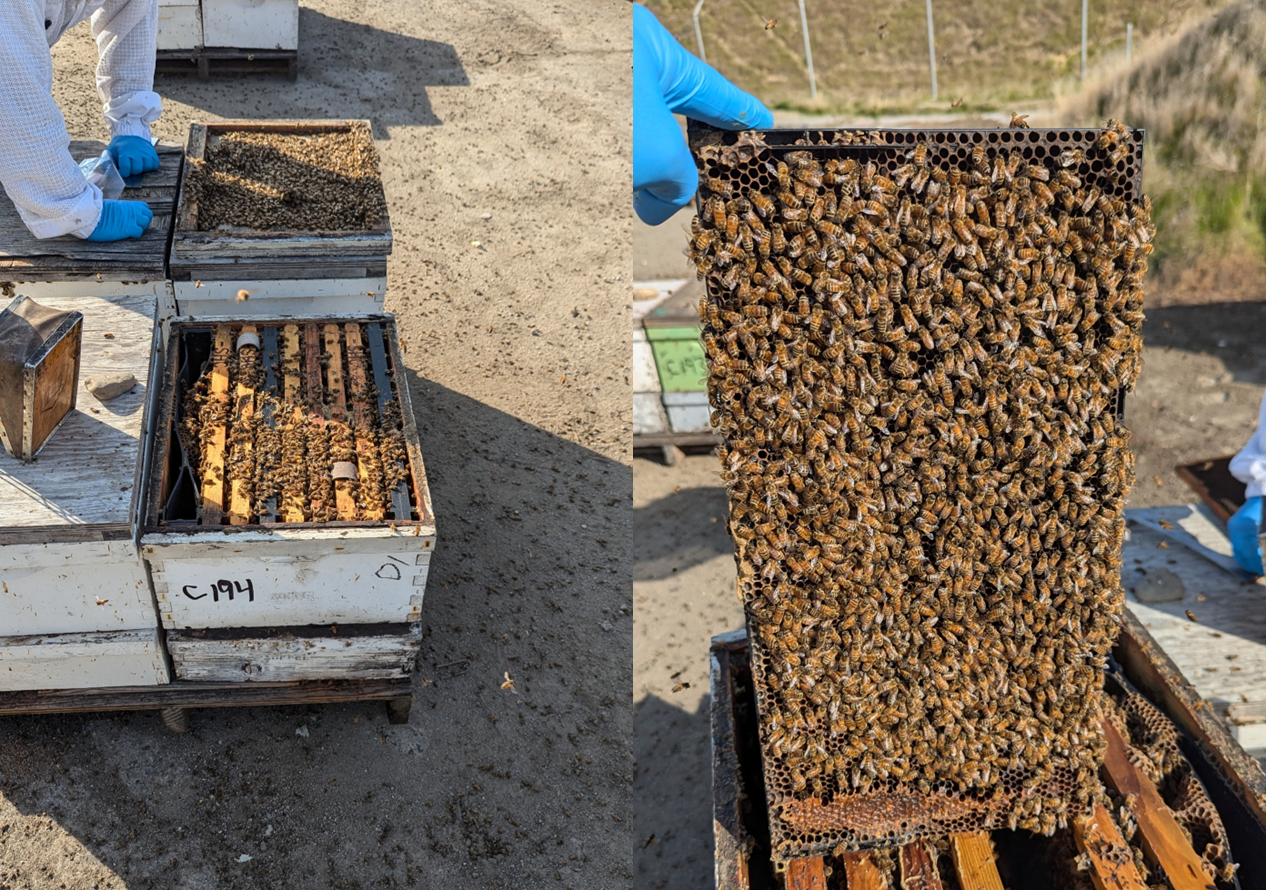
**

**Fig D.** A typically strong colony, showing coverage across all internal frames, and dense coverage of adult bees on hive surfaces.

1. **United colonies that continue to dwindle**

Beekeepers will combine similarly weak colonies with the expectation that the combined colonies will perform better than their component parts (*hereafter* ‘combined colonies’). We sampled combined colonies in two of the six operations in this study. The success of those colonies varied widely. In Operation 3 combined colonies faired poorly with 15 out of 18 combined colonies losing bees between the time of their unification and our inspection (83.3%). In Operation 4 a similar result was observed with 46.2% of combined colonies evidently losing bees (6/13). Deductions on bee loss were largely qualitative and made by deducting the duration between beekeeper unification of colonies, and the condition of the colony at our inspection.


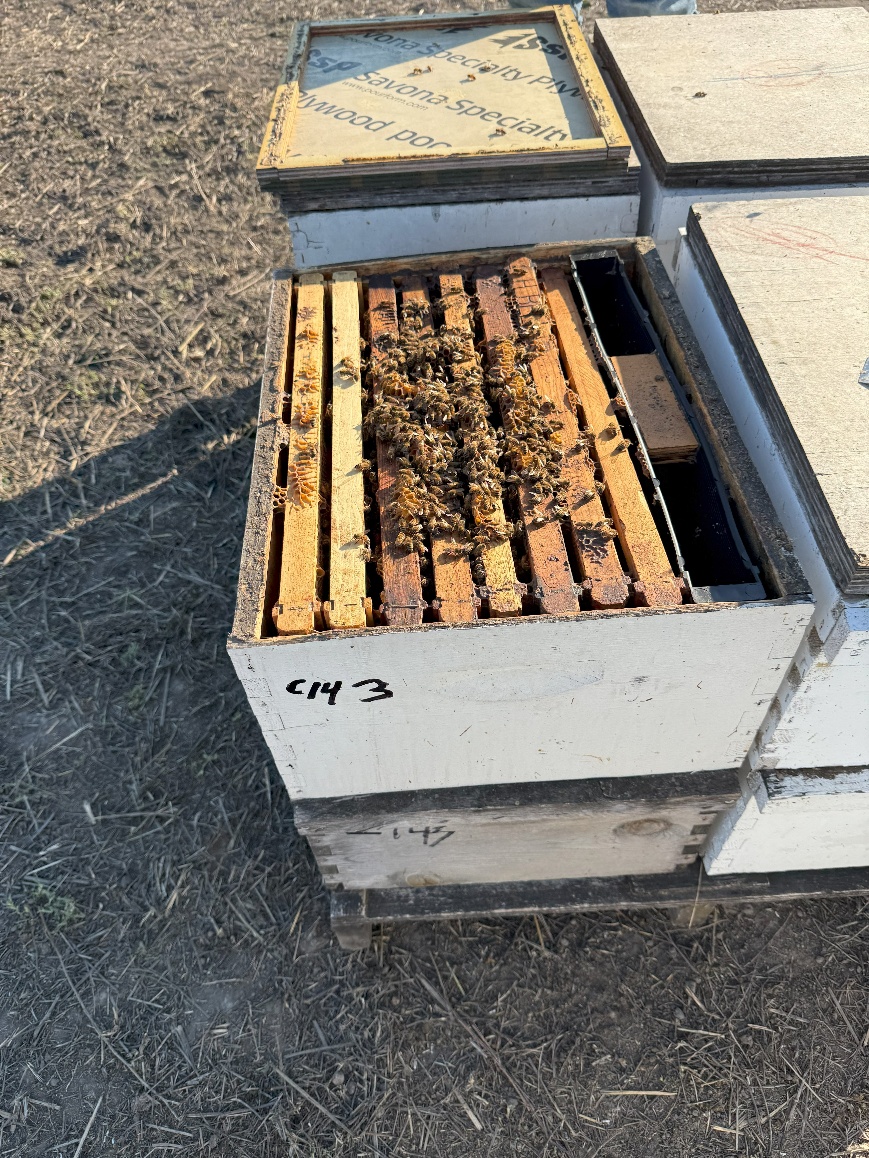


**Fig E.** A colony which continued to dwindle after uniting with another colony.


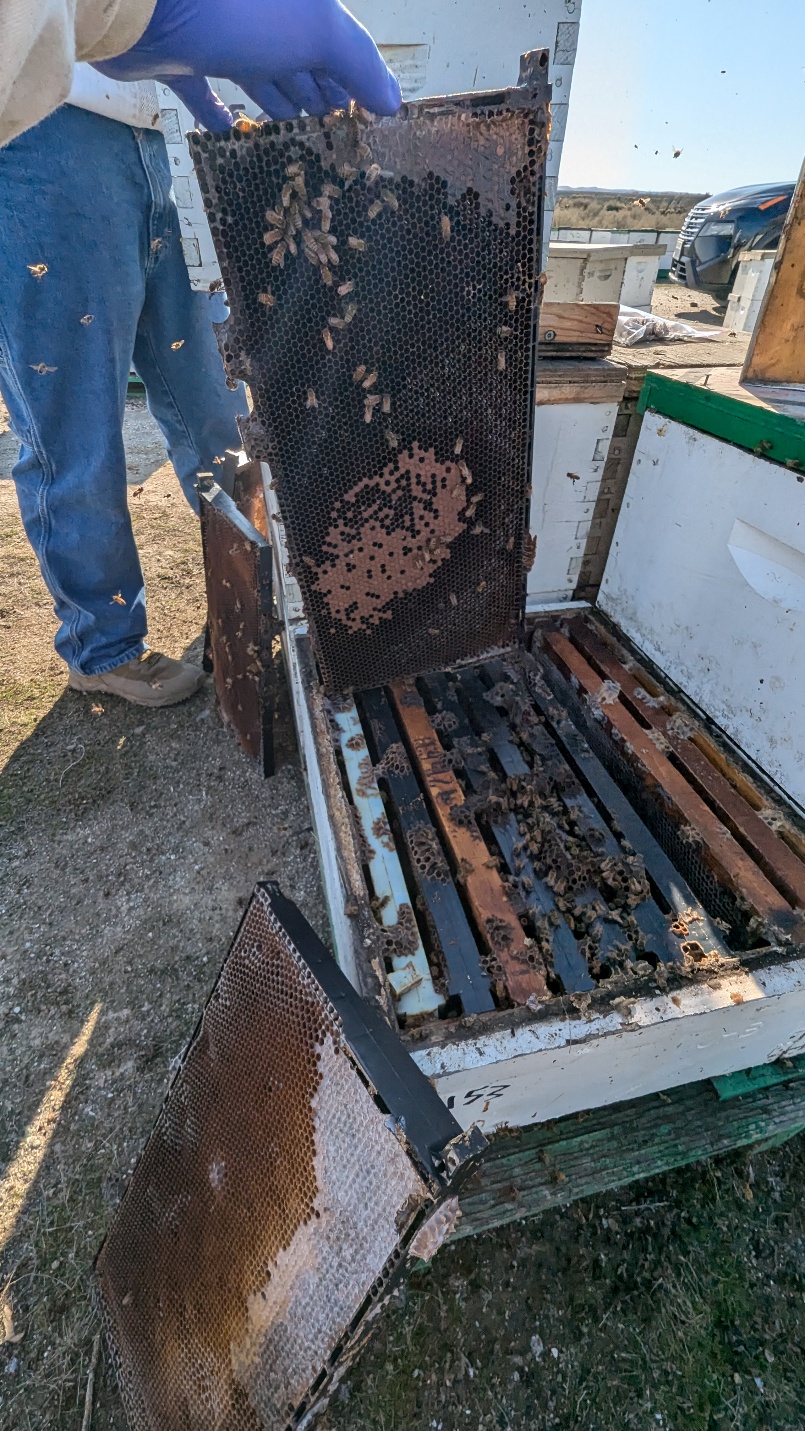


**Fig F.** A typically “dwindled” colony which was recently combined. This colony lost approximately 6 frames of adult bee coverage between January 21^st^, 2025 when it was inspected by the beekeeper, and January 28^th^, 2025 when inspected by our research team.

1. **Survivorship bias**

Operation 5 overwintered colonies in climate-controlled ‘shed’ storage. Colonies were transported to CA from storage on Wednesday January 29^th^, and were sampled on the morning of January 31^st^. The beekeeper removed dead colonies, but left the dead bees from those colonies, including undisturbed bottom board debris. As a typical description, surviving and perished colonies had hundreds to thousands of recently dead bees exuded in the front of colonies. Dwindled colonies were typically small, with a low density of adult bees covering several frames. Brood was lacking or represented by a small patch, though that description is typical for colonies immediately out of indoor storage. Strong colonies were typically robust, with dense adult bee coverage. Brood was typical of colonies recently released from indoor storage. Perished colonies had *Varroa* visible on bottom boards and/or in the mass of adult bees outside entrances. Figure 5 is a typical representation. Every pallet bottom board inspected for debris had recoverable *Varroa*, except for two (5/7). The number of *Varroa* varied, but were never quantified. Several colonies visibly had hundreds of dead mites, while others had fewer (Figure 6). We contrasted this qualitative observation between perished and surviving colonies as surviving colonies had few observable Varroa while remnants of perished colonies, when inspected, had many.


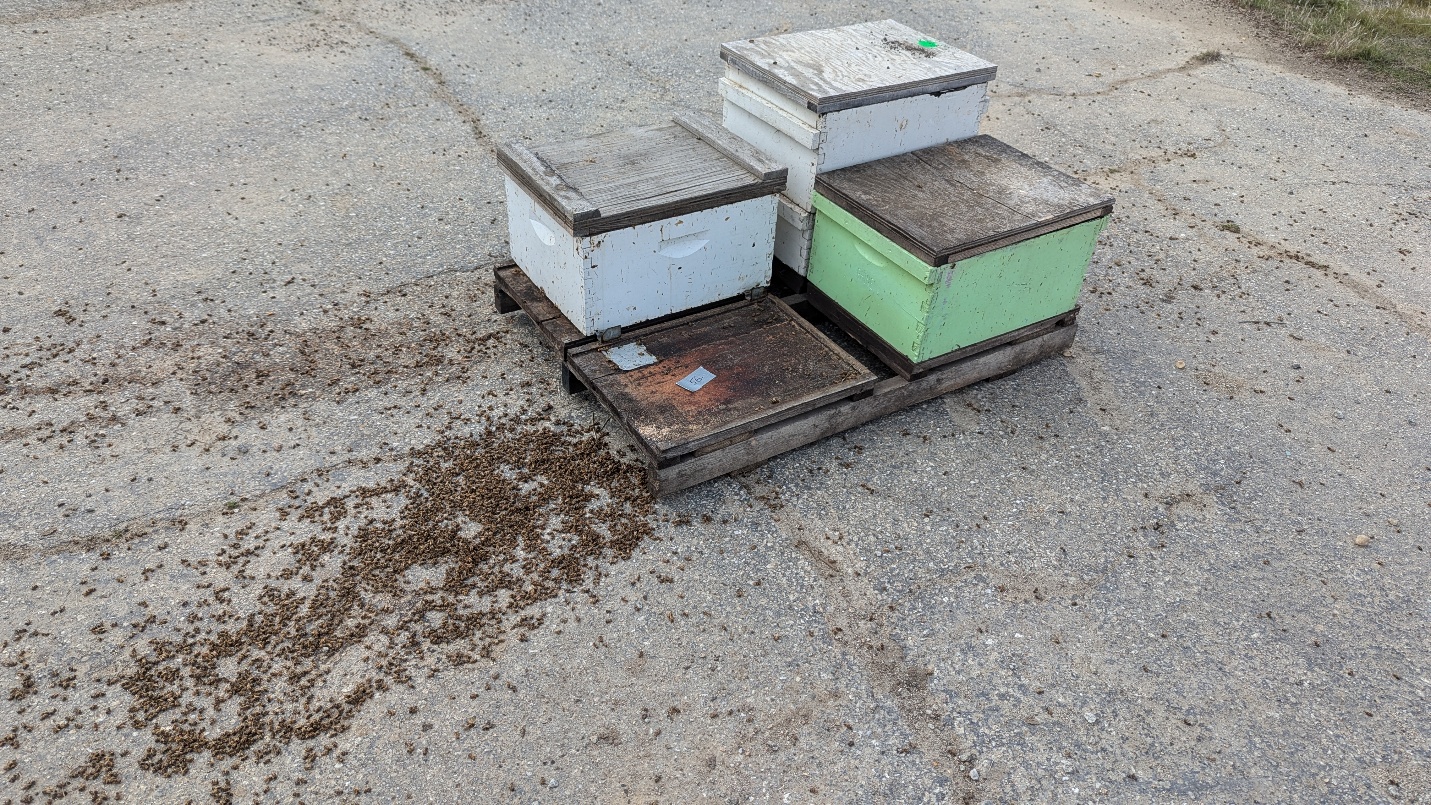


**Fig G.** Remains of a perished colony includes a mass of recently dead bees and pallet board debris.


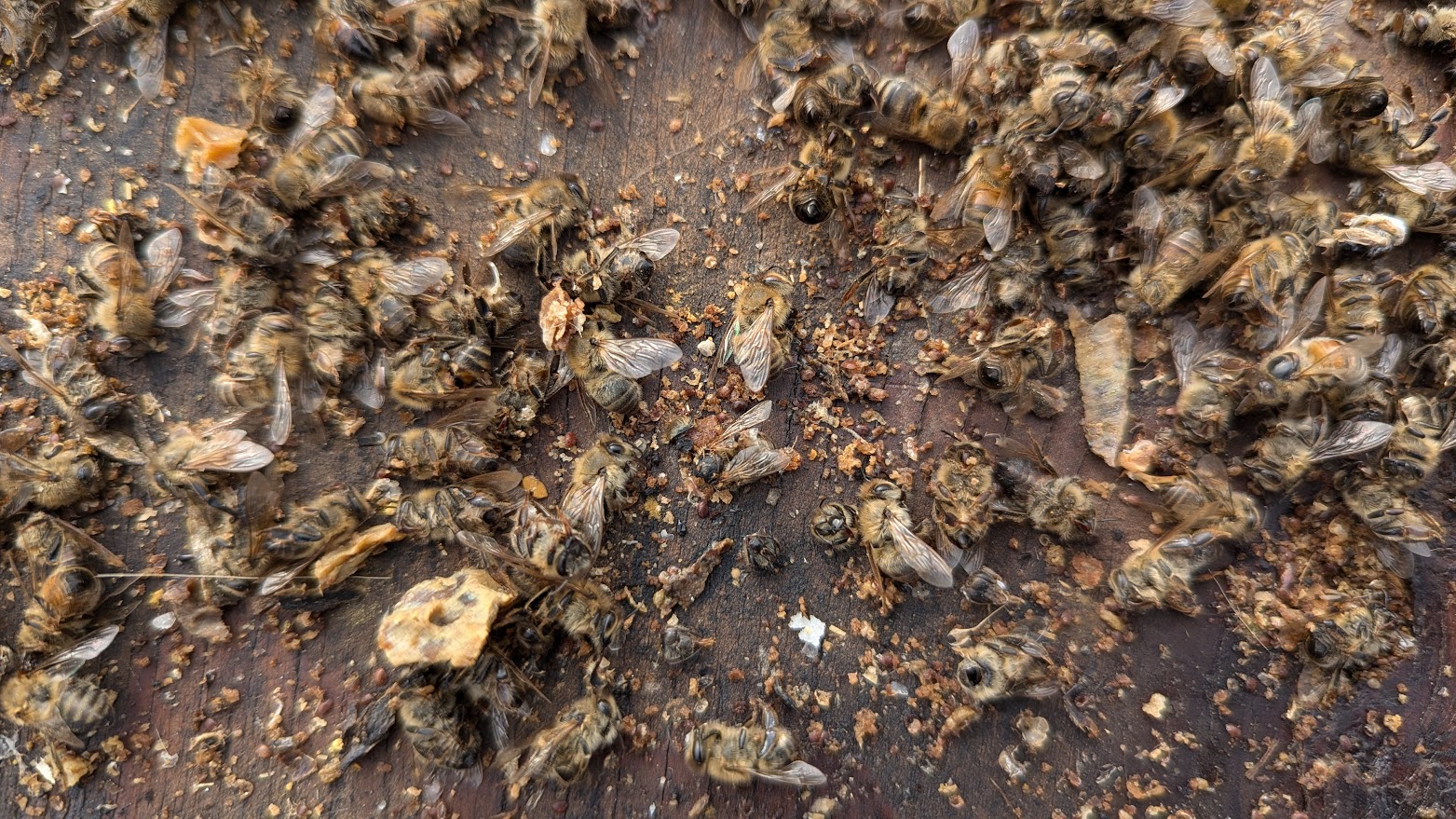


**Fig H.** An up close picture of pallet board debris, featuring numerous *Varroa.*

1. **Symptomatic bees**

Symptomatic bees were observed leaving from colony entrances. They were unable to fly, and instead crawled on the ground. Some became fully immobilized, appearing dead but we reactivate when warmed in the hand. Others would be able to make a short, downward flight from the colony entrance, and then crawl away from the colony. Commonly, bees would quake or shake in place or as they mobilized away from the colony. Some would climb vegetation (if present) and stay adhered to the stems. Some bees engaged in erratic movements after egressing the colony, without seemingly the ability to mobilize in a forward motion.

The rate of bees leaving colonies was unknown. In Operation 1 a carpet of degraded, perished bees, variable in size, was present in front of colony entrances. However, it is unclear if the mass of bodies accrued over a short or long duration. At Operation 2 bees could be observed leaving affected colonies every minute. Stooping over the colony entrance, one could watch morbid bees intermittently egressing. Sometimes an undertaker bee would carry out a symptomatic bee. In Operation 2 foragers, with corbicula filled with fresh pollen, were observed to lose flight and land around their colonies, unable to finish flying into the entrance.


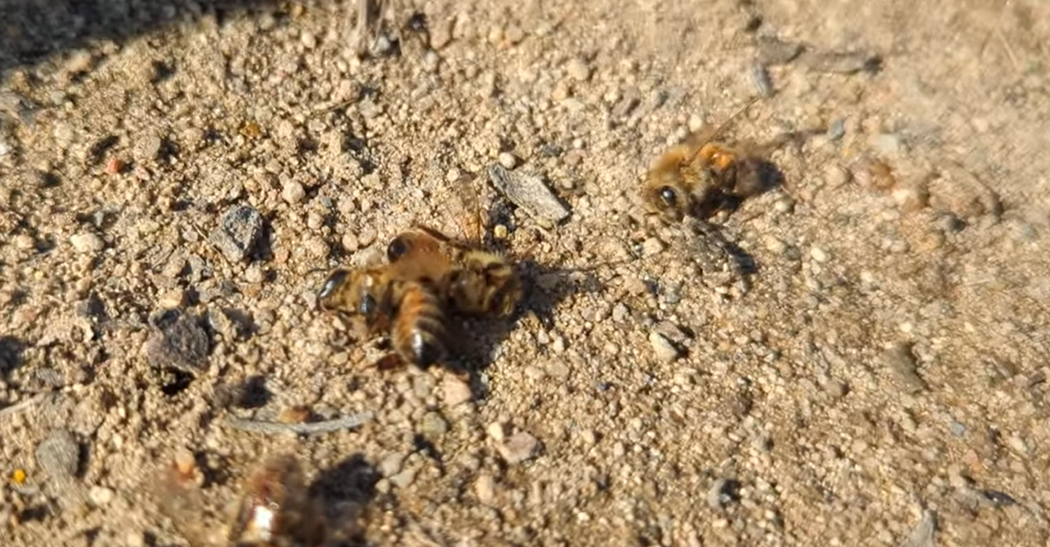


**Fig I.** Immobilized adult worker bees, having recently egressed from their colony.

**List of Legends - Supplementary**

**Table A.** Primer names and associated sequences for qPCR detection and relative quantification of common honey bee pathogens.

**Table B.** Primer names and associated sequences for qPCR detection and absolute quantification of four pathogens in experimental inoculum.

**Table C.** Dilution factor of four independent inoculum (representing portion of pupa) and copy number per ul of inoculum

**Table D:** B-Actin and viruses screened in inoculums.

**Table E:** Prevalence of pathogens found in asymptomatic and symptomatic bees.

**Table F:** Inoculums and survivorship of adult bees

**Fig A:** Pathogen detections by colony condition. **A)** Strong, **B)** Medium, **C)** Weak.

**Fig B:**Amplification of DWV-B and ABPV targets in individual, injected pupae at time zero, 36 and 60 hours post injection. A subset of pupae were sacrificed at each time point, and used as a representative for viral infection for that specific timepoint.

**Fig C:** A typical ‘dwindled’ colony: honey stores largely intact, queen present, a patch of brood, and adult bee abundance lacking *(Photo Lamas)*

**Fig D:** A typically strong colony, showing coverage across all internal frames, and dense coverage of adult bees on hive surfaces. *(Photo Lamas)*

**Fig E:** A colony which continued to dwindle after uniting with another colony. *(Photo Lamas)*

**Fig F:** A typically “dwindled” combined is picture above. This colony lost approximately 6 frames of adult bee coverage between January 21^st^, 2025 when it was inspected by the beekeeper, and January 28^th^, 2025 when inspected by our research team. *(Photo Lamas)*

**Fig G**: Remains of a perished colony includes a mass of recently dead bees and pallet board debris. *(Photo Lamas)*

**Fig H**: Pallet board debris, featuring numerous *Varroa. (Photo Lamas)*

**Fig I:** Immobilized adult worker bees, having recently egressed from their colony. *(Photo Lamas)*
