# Supplementary material for: Viruses and vectors tied to honey bee colony losses
Source: PLoS Pathog. 2026 Feb 23;22(2):e1013939. doi: 10.1371/journal.ppat.1013939 (PMC12948310; doi:10.1371/journal.ppat.1013939)
Supplement: Attachment — Submitted filename: ReviewerResponse091925_LamasEvans.docx [file ppat.1013939.s003.docx]

PPATHOGENS-D-25-01256
Viruses and vectors tied to honey bee colony losses
PLOS Pathogens

Dear Dr. Lamas,

Thank you for submitting your manuscript to PLOS Pathogens. After careful consideration, we feel that it has merit but does not fully meet PLOS Pathogens's publication criteria as it currently stands. Therefore, we invite you to submit a revised version of the manuscript that addresses the points raised during the review process.

Please submit your revised manuscript within 60 days Aug 16 2025 11:59PM. If you will need more time than this to complete your revisions, please reply to this message or contact the journal office at plospathogens@plos.org. When you're ready to submit your revision, log on to https://www.editorialmanager.com/ppathogens/ and select the 'Submissions Needing Revision' folder to locate your manuscript file.

Please include the following items when submitting your revised manuscript:

* A rebuttal letter that responds to each point raised by the editor and reviewer(s). You should upload this letter as a separate file labeled 'Response to Reviewers'. This file does not need to include responses to any formatting updates and technical items listed in the 'Journal Requirements' section below.
* A marked-up copy of your manuscript that highlights changes made to the original version. You should upload this as a separate file labeled 'Revised Manuscript with Track Changes'.
* An unmarked version of your revised paper without tracked changes. You should upload this as a separate file labeled 'Manuscript'.

If you would like to make changes to your financial disclosure, competing interests statement, or data availability statement, please make these updates within the submission form at the time of resubmission. Guidelines for resubmitting your figure files are available below the reviewer comments at the end of this letter.

We look forward to receiving your revised manuscript.

Kind regards,

Adam G Dolezal
Guest Editor
PLOS Pathogens

Sonja Best
Section Editor
PLOS Pathogens

Sumita Bhaduri-McIntosh
Editor-in-Chief
PLOS Pathogens
orcid.org/0000-0003-2946-9497

Michael Malim

Editor-in-Chief
PLOS Pathogens
orcid.org/0000-0002-7699-2064

**Additional Editor Comments:**

As the authors can see from the reviewers, the referees felt this was important work and will be impactful, likely receiving significant engagement and scrutiny from beekeepers and the public. However, the current form of the manuscript seems rushed, has some attention to detail issues, and appears to overstate some of the results.

Please address reviewer comments and pay special attention to the following

-lack of varroa mite infestation data and amitraz application data

- Mite infestation numbers, talk to Frank re: including more acaricide data?
  -comparison to healthy colonies to contextualize the virus loads and justify the argument that the observed levels are higher than ‘normal’

1) *all prior surveys*
2) *any other western US results we have?*

-attention to detail on figures and figures legends; legends are very sparse, figures are hard to read, and there are several inconsistencies between the figures and results.
-The reviewer comments focus primarily on the virus/pathogen aspects of the study, but some touched on the amitraz resistance point and I want to highlight these. For example, the manuscript refers to ‘universal’ presence of a genetic marker associated with miticide resistance. However, the sample size is quite small, the sampling methods are not clear, and no Varroa load or amitraz treatment data is provide, making it very difficult to make such a comprehensive claim.


**Journal Requirements:**

1) Please ensure that the CRediT author contributions listed for every co-author are completed accurately and in full.
At this stage, the following Authors/Authors require contributions: Zachary S Lamas, Frank Rinkevich, Andrew Garavito, Allison Shaulis, Dawn Boncristiani, Elizabeth Hill, Yan Ping Chen, and Jay Evans. Please ensure that the full contributions of each author are acknowledged in the "Add/Edit/Remove Authors" section of our submission form.
The list of CRediT author contributions may be found here: [https://journals](https://journals/).plos.org/plospathogens/s/authorship#loc-author-contributions

2) We ask that a manuscript source file is provided at Revision. Please upload your manuscript file as a .doc, .docx, .rtf or .tex. If you are providing a .tex file, please upload it under the item type u2018LaTeX Source Fileu2019 and leave your .pdf version as the item type u2018Manuscriptu2019.

3) Please provide an Author Summary. This should appear in your manuscript between the Abstract (if applicable) and the Introduction, and should be 150-200 words long. The aim should be to make your findings accessible to a wide audience that includes both scientists and non-scientists. Sample summaries can be found on our website under Submission Guidelines:
<https://journals.plos.org/plospathogens/s/submission-guidelines#loc-parts-of-a-submission>

***Done**

4) We do not publish any copyright or trademark symbols that usually accompany proprietary names, eg ©,  ®, or TM  (e.g. next to drug or reagent names). Therefore please remove all instances of trademark/copyright symbols throughout the text, including:
- ® on pages: 5, and 8
- TM on pages: 4, 5, and 8.

***Done**

5) Please upload all main figures as separate Figure files in .tif or .eps format. For more information about how to convert and format your figure files please see our guidelines: 
[https://journals](https://journals/).plos.org/plospathogens/s/figures

6) We have noticed that you have uploaded Supporting Information files, but you have not included a complete list of legends. Please add a full list of legends for your Supporting Information files after the references list.

7) Some material included in your submission may be copyrighted. According to PLOSu2019s copyright policy, authors who use figures or other material (e.g., graphics, clipart, maps) from another author or copyright holder must demonstrate or obtain permission to publish this material under the Creative Commons Attribution 4.0 International (CC BY 4.0) License used by PLOS journals. Please closely review the details of PLOSu2019s copyright requirements here: PLOS Licenses and Copyright. If you need to request permissions from a copyright holder, you may use PLOS's Copyright Content Permission form.
Please respond directly to this email and provide any known details concerning your material's license terms and permissions required for reuse, even if you have not yet obtained copyright permissions or are unsure of your material's copyright compatibility. Once you have responded and addressed all other outstanding technical requirements, you may resubmit your manuscript within Editorial Manager.

Potential Copyright Issues:
i) Please confirm (a) that you are the photographer of 4, and S1, or (b) provide written permission from the photographer to publish the photo(s) under our CC BY 4.0 license.

***Photos by Z. Lamas**
8) We note that your Data Availability Statement is currently as follows: "Data is available in accompanying Supplementary File.". Please confirm at this time whether or not your submission contains all raw data required to replicate the results of your study. Authors must share the “minimal data set” for their submission. PLOS defines the minimal data set to consist of the data required to replicate all study findings reported in the article, as well as related metadata and methods ([https://journals](https://journals/).plos.org/plosone/s/data-availability#loc-minimal-data-set-definition).
For example, authors should submit the following data: 
1) The values behind the means, standard deviations and other measures reported;
2) The values used to build graphs;
3) The points extracted from images for analysis..
Authors do not need to submit their entire data set if only a portion of the data was used in the reported study.
If your submission does not contain these data, please either upload them as Supporting Information files or deposit them to a stable, public repository and provide us with the relevant URLs, DOIs, or accession numbers. For a list of recommended repositories, please see [https://journals](https://journals/).plos.org/plosone/s/recommended-repositories. 
If there are ethical or legal restrictions on sharing a de-identified data set, please explain them in detail (e.g., data contain potentially sensitive information, data are owned by a third-party organization, etc.) and who has imposed them (e.g., an ethics committee). Please also provide contact information for a data access committee, ethics committee, or other institutional body to which data requests may be sent. If data are owned by a third party, please indicate how others may request data access.

**Reviewers' Comments:**

Reviewer's Responses to Questions

**Part I - Summary**
Please use this section to discuss strengths/weaknesses of study, novelty/significance, general execution and scholarship.

Reviewer #1: Editors,

The manuscript entitled, “Viruses and vectors tied to honey bee colony losses” by Lamas et. al Evans describes the prevalence and abundance of pathogens (with a focus on viruses)
The authors used pathogen specific PCR to test for 13 pathogens and qPCR to examine the relative quantification of a subset of these pathogens, in honey bee samples collected from several beekeeping operations experiencing high levels of colony losses (including those that are typical of dwindling colonies or more unusual deaths, as nicely described in the supplemental information, though the section on survivorship bias requires editing). The colony level data pathogen levels did not reveal and striking differences in colonies that were weak, medium, and strong (Fig. 1), while the levels in individual morbid vs. asymptomatic bees differed for DWV-B virus (most striking in Fig. 2) and ABPV. In addition, the team determined that Varroa destructor parasites and viral vectors infesting these colonies had a gene/allele associated with miticide resistance (i.e., susceptible or resistant allele of the octopamine receptor (Octβ2R). Overall, the manuscript provides a good description of the data, though it seems some methods, technical aspects and details should be improved and/or clarified prior to publication in PLOS Pathogens. These details may not impact the overall findings, but they would improve the overall quality of the manuscript.

Reviewer #2: This study provides some much-needed empirical data on the current U.S. colony loss situation. The data are valuable; however, in many cases the origins of the samples and other methodological details are insufficiently described. In addition, I have several major concerns that might result in the manuscript being better presented as a survey rather than having confidently (as stated at line 425) identified the causal factor(s) underlying losses. The experimental work on which this causality relies involves testing inocula from just three symptomatic bees on recipient bees from just one colony, which is not sufficient for such a bold claim, in my opinion.

Up to the authors, but I think the discussion would be strengthened if potential socioeconomic factors are described. We are often quick to point to a new disease or poison, but some big-time beekeepers in California have brought up the fact that beekeepers have been feeling the economic squeeze due to labor costs, reduced pollination revenue, etc., which inevitably trickles down to reduced investment in keeping their colonies healthy. I think they are right, that the economic situation shouldn’t be ignored. Just for your consideration.

Reviewer #3: This is an important and time-sensitive paper that provides data on the potential causes of recent high honey bee colony mortality in the USA. In general, it is well-written, although some sections felt rushed, and more attention to detail is required before publication. Given the level of scrutiny and interest it is likely to generate, the authors need to take the time to be precise, flesh out additional required details, and place their results in a broader context. To this end, I have made suggestions (below, in the specific comments section) for the authors to consider.

Reviewer #4: In this study, the authors sampled the many colonies were still actively collapsing in January, 2025, by screening pooled and individual samples for levels of known honey bee pathogens and parasites. The limitation of this study is that they focused not only on known honey bee pathogens and parasites BUT they did not consider the variants that their methods likely missed. At a minimum, this limitation was not discussed at all, especially based on the more advanced technologies available. Both Deformed wing virus strains A and B was identified as possible agents on concern. However, the data as presented does not totally agree with this conclusion (see specific points raised below). Along with DWV, Acute bee paralysis virus, was also found at unusually high levels, either in pooled colony samples or in individual bees exhibiting shaking behaviors and morbidity. The mortality experiments confirm what was previously known about the aggressive and pathological nature of the AKI complex viruses. Only DWV-B could be tested their mortality experiments, and it too show negative effects on bee health (when injected) but not as bad as ABPV.
The "direct collections of morbid bees provide a superior diagnostic for causal viruses" is an overstatement especially given that the morbid bees came form a small subset of teh colonies screen.

**Part II – Major Issues: Key Experiments Required for Acceptance**
Please use this section to detail the key new experiments or modifications of existing experiments that should be absolutely required to validate study conclusions.

Generally, there should be no more than 3 such required experiments or major modifications for a "Major Revision" recommendation. If more than 3 experiments are necessary to validate the study conclusions, then you are encouraged to recommend "Reject".

Reviewer #1: Points to clarify or address before publication include:

1. Figures 1 and 2 - It may be nice to visualize both of those figures as clustered bar-graphs that separate the designations (e.g., weak, medium, or strong or morbid vs. asymptomatic) to make the data included the means easier to visualize). Including these additional representations of the data as supplemental figures may be best.

***This is an excellent recommendation. We made a figure as suggested, and will refer to it in Supplementary. Readers who wish to visualize, as you point out, will be able to. We have elected to keep pathogens in alphabetical order. However, if reviewers request grouping we will accommodate that.**

2. Fig. 1 – Colony level results, given the natural variability in virus abundance in honey bee colonies the sample sized in this study (i.e. 72 weak and 41 strong colonies) may have been too small to detect differences, unless those differences were large in scale or particular pathogens were only present in weak colonies, etc.. While the authors were limited in the number of samples that could feasibly be obtained during an acute loss event, the authors should comment on this in the Discussion section of the manuscript (see and consider citing - Faurot-Daniels et al PONE 2020, power analysis, virus dynamics).

***This is a good point, one reason for hand-picking symptomatic and healthy bees was just this, worry about the variance within and across colonies and resultant noise. Given the common use, by ourselves and others, of 50-bee samples as an indicator of colony status or risk, the Faurot-Daniels paper and our own apparent lack of signal are sobering, and we have added a citation and discussion of the former in the current discussion section. Another reason, arguably, was that collections of 50-bee cohorts at a set timepoint could be subject to representation error. To address this in part, we analyzed a smaller longitudinal study from Maryland, after which it was revealed that colonies with high levels of both common DWV strains, but not ABPV, were overly prone to collapse. This result gives more confidence that these viruses can be predictive, even from group samples collected and analyzed in an identical way. Our analysis of individual bees with healthy and sick appearances also suggests that viral loads are worthy metrics for bee health even at a modest scale.**
3. Colony level mite data should be included, if available. If not, a Table indicating what colonies the mites came from and number sampled, etc. should be included in the results or as supplemental material.

***Since colonies were in various states of decline, some almost completely, we felt that mite counts as a percentage of worker bees would be inflated in the collapsing colonies, fitting the model of mite-induced mortality but arguably subject to bias as well**

4. It would be good for the authors to include more information on miticide resistance (i.e., the percent resistances that is associated the octopamine receptor
susceptible or resistant allele), though some information is included in the Discussion.

***We have expanded this in the discussion, namely pointing out the numbers behind phenotypic and molecular cues for resistance as stated in Rinkevich et al., 2023**

5. Line 282-283 and in relevant section of Discussion, since the authors only distinguish DWV strains by qPCR of a relatively small region of the virus genome (~ 130 nt) of a 10,000+ nt genome, it may be that the viruses they are detecting and describing are recombinant strains (i.e., not really DWV-A or DWV-B). Sequencing data would be informative, thought it would only provide a consensus sequence that represents all of the viruses circulating in the bee at the time of sequencing, and therefore, to really distinguish virus strains long read sequencing data would be required. The authors should discuss this in the text.

***This is a good point and an active field, we have expanded the citations to methods and arguments for this strategy and feel it is complimentary. In defense of the described single-point PCR analyses, if these targeted sections were not important we feel we would not have seen a strong discrepancy between morbid and healthy bees based on qPCR.**

6. Lines 20-21, and throughout the manuscript the authors refer to usually high levels of virus. To facilitate comparison with other studies, and to ensure accuracy of virus abundance – based on RNA copies, additional sample details (i.e., amount of RNA obtained, virus RNA copies per x ng total RNA) and clarification of the relative qPCR data, which seems to be presented as deltaCt data (i.e., relative to honey bee actin RNA). The authors should cite comparable are cited, D'Alvise et al Ecology and Evol. 2019, and others.
***Good points and we were reflecting this based on similar dCT analyses, D’Avise et al is a great addition and is now cited in the Discussion D'Alvise, P., V. Seeburger, K. Gihring, M. Kieboom, and M. Hasselmann. 2019. Seasonal dynamics and co-occurrence patterns of honey bee pathogens revealed by high-throughput RT-qPCR analysis. *Ecology and Evolution* 9:10241-10252.**

7. While recognizing that it may not be possible to test for all common honey bee pathogens in all samples, it seems it is important for the authors to test for additional pathogens, at least in select pooled samples. Specifically, it would be good for the authors to test for the (1) bacterial pathogens that cause foulbrood diseases (i.e., P. larvae and M. plutonius), while those may be diagnosed in the field, it seems important to also test for them using more sensitive molecular tests; (2) AmFV, a DNA virus that has recently been described as abundant in the US (Cox-Foster- unpublished), or an Iridovirus that has been discussed by beekeepers, etc. and was investigated during previous high colony loss events in the US (e.g. Cornman et al 2012 PONE). I suggest that the authors do additional testing of pooled cDNA samples (e.g., each operation / sample date or healthy vs morbid bees, etc..).
***We are carrying out a metagenomic analysis of pooled samples from these events. Nevertheless, of the proposed targets above there were no signs of widespread brood disease for the contagious bacterial pathogens. We do not have current unpublished data from Cox-Foster but in our prior efforts, including Cornman et al, 2021, AmFV was not common nor was it tied to rapid colony loss. The proposed iridovirus came from a flawed proteomic analysis (Knudsen GM, Chalkley RJ (2011) The Effect of Using an Inappropriate Protein Database for Proteomic Data Analysis. PLoS ONE 6(6): e20873. https://doi.org/10.1371/journal.pone.0020873) and this too has not been found widely nor has it been linked to disease, while Alvise et al showed a 0.4% positive rate for this class of viruses in their 2019 study/above, Nevertheless, we now present data for two additional suggested targets: AmFV and the tracheal mite *Acarapis woodi*. Neither was found in this sample set using our primers.**

8. Likewise, it would have been good for the authors to do some untargeted analysis (i.e., high throughput sequencing of select pooled samples), which may reveal new pathogens or new/different viral strains that may prevalent in this sample cohort.
***We acknowledged the merits of metagenomic sequencing in the original text, and cite a recent paper (Hesketh-Best et al.), that is the first to do this at a large scale (i.e, dozens of samples while highly cited prior work, e.g., Cox-Foster et al., was based on a single pairing of a ‘collapsing’ pool of RNA and a ‘healthy’ pool). Applying these techniques to individual colony samples or apiaries, when feasible, and connecting with colony status, will be insightful. As above, we would love to do that and also, of course, encourage others to collect and compare in this way from similar events. We have had institutional setbacks getting this done in a timely way. In this analysis, we have found strong signals, for symptomatic bees, among the ‘usual suspects’ indentifiable by established PCR primers, but that does not rule out other important causes.**

9. While sequencing and analyzing pooled samples may be beyond the scope of this article for the sample cohort, the authors should perform sequence analysis on their inoculum. In addition, the description of the inoculum is confusing and should be improved (i.e., “bee equivalent microliters”). As I understand it the authors, made inoculum from bee samples that were ground in liquid nitrogen and subject to several freeze/thaw cycles, and then suspended in buffer and filtered. The team has several inocula described as “CV-3 -4, -5, -7”, which these names may refer to specific samples in the cohort, for the purposes of the manuscript it would be clearer to call them inoculum #1-4, or A, B, C, D.  **We changed these to inoculums A,B,C,D.**

***We acknowledge that sequencing to identify viral targets and recombinants in these inocula will be deeply interesting. We hope this interest does not detract from our findings presented here. DWV-B, DWV-A and ABPV were identified in high levels in the inocula made from individual dying bees procured before perishing during a severe loss event. The purposes of the longevity impact experiment using these inocula were to see if 1) Morbid bees collected in the field had high levels of virus 2) Could viruses with known virulence be identified in those bees ansd most importantly 3) Does material from those individual dying bees cause mortality and morbidity, i.e. recreate the losses from the field in the laboratory and implicating viruses rather than chemical or non-viral microbial causes as key triggers. This gave us strong epidemiological evidence that viruses, arguably a combination of the three aforementioned, were causative in the adult bee losses. We do believe that these viruses represent the most parsimonious answer for the losses given the collective data in hand, while also hoping for new insights once we are able to accomplish sequencing. We also felt the current results were urgent and informative and feel that scope would be quite different than the focused scope of this work. We have recoded the inocula IDs alphabetically for clarity.**

Table S3 described the copy number of DWV-A, DWV-B, and ABPV in the inoculum as per ul of inoculum, which may be fine - but since the efficiency of RNA extraction from dilute virus stocks etc. varies, and to ensure RNA was adequately extract from all samples, etc. the copy number should be express as per X ng of RNA/cDNA in the qPCR reaction, which is what is measured. The authors can provide a per lysate copy via converting back to ul lysate, if they know that total amount of RNA isolated from a given amount of lysate. In short, it is critical to this study that the virus inoculum/lysate is well characterized and that the numbers are accurate; as currently presented it is not possible to evaluate this, the inclusion of an excel table including inoculum, RNA quantity, qPCR Ct values, standard curve values, etc. should be included as Supplementary information.

***We debated these various strategies before coming up with the method we used to categorize the four inocula (e.g., using CT values, and a synthetic known reference). Most importantly, we filtered out host cells in the inoculum prep, and feel this likely compromised the use of host b-actin as a control for a statistic like dCT. We could also use raw RNA as a metric but that would reflect both the viruses that passed through intact and any free RNAs from lysed host or microbial cells, a ratio we really could not control. Instead of assuming this would be absent, or equal, across inocula, and in keep with our main goal ( inference of how many copies of viable viruses were given to bees) we feel that using CT directly for the target viruses was best.**

It is also not clear if the data in Table S3 represent six independent isolations of virus RNA from diluted stocks, which were qPCR – which is unlikely give that the numbers are just serial dilutions. What samples were assessed? Maybe undiluted inoculum?

Table S3: Thank you we can clarify that. There were only four inocula, each diluted across a six-fold range. The new shading on this Table should make this more clear.

It is not clear what “representing a portion of pupa”, nor “bee equivalents” refers to, it would be best to include a definition and/or description of the equation for “bee equivalents” the authors are referring to. It likely makes sense to their team, and may be accurate, but it may not be clear to other scientists/readers of this article.

Thank you. We have defined ‘bee equivalents’ more clearly now, apologies for this oversight.

To better compare the inoculums, it would be useful for the reader to know the total RNA amount obtained from each inoculum (it would be best to have isolated RNA from 3 dilutions per inoculum to ensure that RNA isolation was uniform, since this essential for absolute quantification), this would also facilitate better comparison of the inoculum.
***See below re: rationale for using ‘bee-equivalent’ inocula and the effort to compensate for titer differences by using 100-fold ranges in dilutions, 1/10,000 for CV-5.**
It is particularly important to know if RNA was sufficiently extracted from inoculum CV-7, if not – it would not be possible to detect pathogens. While inoculation data indicate that this inoculum may be pathogen-free, this should be supported by additional data indicating that this wasn’t a failure of RNA extraction (since host RNAs should still be present in these lysates).

***As above, we do not know how much host/cpntrol RNA fmro lysed cells would have made it through the0.2 uM filter. To make sure there was indeed some substance, though, we have provided nanodrop data in supplementary materials as requested. To cover any variance across inocula in pathogencity caused by titers, we used three concentrations of inoculum in the longevity trial. Each adult bee received either 10^-4^, 10^-5^, 10^-6^, bee equivalents. (We have now fully defined this in methods). Each concentration represents 1/1,500^th^, 1/15,000 or 1/150,000^th^ of the original bee used to make the inoculum. Secondly, we delivered the inoculum as portions of a bee so that the mortality induced can be compared across inocula relative to their dilution factor. The second metric we used to quantify the amount delivered to each bee was to perform absolute quantification to describe how many GE per ul of inoculum.**

PCR or qPCR for host B-actin in the sample samples from which virus quantification data was obtained should be done for all inoculum that were used in this study. If original inoculum is not available, then new aliquots could be assessed if qPCR analyses were done for all viruses and B-actin (i.e., from the same RNA extraction).

***As above, host b-Actin transcripts are unreliable for quantifying RNA in these inocula.**

10. Table S5 – consider changing “Concentration (Bee Equivalent)” to “Dilution Factor” - or better defining this.
*You are correct, we have now better defined this term in justification of its use, in the Material and Methods on lines 170. We did leave the terminology with bee equivalents after defining this term, since this information might be useful for modelers moving forward. Specifically, the new text reads: “Inoculum was diluted as just described, representing a fraction of the original bee source when delivered to adult bees (hereafter bee equivalent, i.e.10^-4,^ is the equivalent of 1/15,000 of the original bee).”

11. Lines 329-332, and Lines 376+ - discussion of results as bee equivalents is not standard and not clear. The authors should improve this, since it is likely they are trying to get across that the pooled bees/pupae that were used to make the virus inoculum had a lot of virus, and in turn could have infected a lot of bees – but without better explanation this is not clear. One virus stock was particularly potent. In Fig. 3, Does the heat-treated inoculum for each trial correspond with the inoculum for each trial (i.e., is it specific)?

*** The extension to number of bees helps explain the substantially higher virulence of this inoculum. We can omit this if it is distracting, but feel by tonming it down in the current version it is clear this is not a literal prediction of threat top bees but a guide to both understanding the described methods and perhaps for future work aimed at ‘ranking’ hazards from virus exposure. Yes, in every case the ‘heat-treated’ controls are for the very inoculum tested in those trials (e.g., CV-5 trials included a CV-5 heat-inactivated control of the 10-4 concentration of that source).**

12. Table S5 – How many independent experiments of the adult bee survivorship studies were performed? It would be best to have multiple, but since there were multiple cages of the most potent inoculum – that is likely sufficient to distinguish that C5 inoculum is more deadly – since data was not obtained from only one representative cage of bees.
***There were two independent trials**

13. Fig. S1 – and other places in the manuscript, it is important that the authors clearly state that T=0, 36, and 60 hrs data are obtained from different animals (i.e., they are not blood draws from the same animals), including additional details in the text, methods, and figure captions would make that clearer. How many pupae per group per timepoint are represented in this figure? Please include a more detailed figure caption.
In addition, the vertical axis labels of this figure appear to be truncated/cut-off in my copy.
 Thank you, indeed each measure of virus load is from an individual ‘once-sampled’ bee. We have added this in the text and figure legend for Figure S1.

14. The representation of the “relative expression of pathogens B-actin-pathogen) using qPCR data requires additional explanation in the methods and figure captions. If I understand correctly, the values on the y-axis are the deltaCt (i.e., B-actin Ct value – pathogen Ct value). If so, it seems the values in Fig. S1 clearly indicate that inoculated pupae have a lot of DWV and ABPV (dCT = 10) compared to those assessed at the zero time point.Assuming B-actin levels in pupae and adults are similar, the dCT values in Fig. 1 seem to rarely exceed “0”, indicating that the viral copy numbers are much less and don’t often get above the levels of actin mRNA in naturally infected bees.
***This is correct, bees were injected directly into the hemocoel with inocula and this led to rapid virus replication, arguably analogous to feeding by infected varroa mites. The Fig. 1 samples reflected a pool of 50 non-treated bees collected directly from hives and hence had, as expected, a lower average viral load (as is often the case on field-collected pools, e.g., Faurot-Daniels, C., W. Glenny, K. F. Daughenbaugh, A. J. McMenamin, L. A. Burkle, and M. L. Flenniken. 2020. Longitudinal monitoring of honey bee colonies reveals dynamic nature of virus abundance and indicates a negative impact of Lake Sinai virus 2 on colony health. *PLoS ONE* 15.)**

Example calculation B-actin Ct value =10, high virus Ct value =5, dCT = 5 (high virus)
Example calculation B-actin Ct value =10, virus RNA same as B-actin Ct value =10, dCT = 0 (virus RNA levels similar to B-actin mRNA levels)
Example calculation B-actin Ct value =10, high virus Ct value =15, dCT = -5 (low virus), negative dCT values
***Yes, these hypothetical calculations reflect our method and are standard. We have fleshed this out better now, thanks for noting this was unclear (current line 148.. needs to show dCT calcs, now has absolute quant.**
If I am understanding Figure 1 correctly, why are there so many very low values for the relative expression of pathogens? Was a sufficient amount of sample assayed to ensure qPCR accuracy?

***Yes, and we thank you for bringing this up. As above, many of the pooled samples had high-CT viral detections indicating extremely low copy numbers. We included these as we felt they were nevertheless non-zero detections. As added to the Supplemental methods, we selected low-level detections for multiple targets and verified accuracy via agarose gels and Sanger sequencing of the qPCR products. Thus we are confident that these reflect extremely low levels of detected virus. Levels for the B-actin controls, now presented fully in the supplements, were uniformly high = low-CT, so the RNA used in these analyses did not vary at the level of specific targeted pathogens nor did it vary across weak and strong colonies.**

15. The authors need to include an excel file with the data the used to generate the figures in this manuscript (i.e, sample name, RNA data (if available), qPCR Ct values, etc.), it is difficult to fully evaluate the qPCR data presented in this manuscript without that information.
**Done**
16. The authors should include details about the linear range of their qPCR assays and their primer efficiency equations.
Done
17. Fig. 2 – The data in this figure is compelling that DWVB levels are strikingly higher in morbid bees compared to asymptomatic bees. How were the bees paired? Did bees have the morphological symptoms associated with DWV infection during development (i.e., crinkled wings, small wings, etc.) or were these just the “generally symptomatic” bees, as described in the paper? Since the abundance of virus in asymptomatic individuals can widely vary, the authors should also show this data as symptomatic individual bee data compared to the “average levels” in asymptomatic bees, and/or using a ranked dCT method, where the largest dCT values from symptomatic bees are paired in a “ranked” manner with the samples that had the largest differences in dCT values for asymptomatic bees, otherwise “pairing” may produce arbitrary results by chance.

***The bees did not exhibit morphological pathologies such as shortened abdomens or crinkled wings as you describe. We tried to limit self-referencing our previous findings in this body of work. However, at this point we will provide a lengthy description of the morbidities and how we believe bees without physical morbidities engaged in aberrant behavior with high levels of DWV-B. This recent work shows that Varroa feed on adult bees at higher frequencies than they feed on developing brood. This is because when Varroa are not imprisoned in a brood cell, they actively move from bee to bee in order to feed. Bees can live for an indefinite period of time after a feeding. However, those feedings when coupled with virus, result in shortened life, and premature death. Additionally, non-parasitized bees can acquire DWV-B through trophallaxis with parasitized nestmates. Thus bees without deformed wings (acquired at eclosion from pupae) nevertheless often carry extremely high and damaging viral titers, often leading to premature death and impaired behaviors while living. We believe our results, when taken together, show that DWV-B infected bees were dying out of colony clusters prior to sampling. This is why DWV-B showed up in only 16.8% of pooled bee samples randomly collected from broodnests, 0% for individual controls, and 95% of dying bees that we screened.**

18. Tables 2A and 2B – it would be good to better explain values in tables – and include corresponding data as a supplemental excel file. The numbers in the table should be standardized to improve readability (i.e., right justified, a single decimal place).

19. Table S4 and within manuscript, please define the target species for “TRYPS” (i.e., what pathogen(s)), it may be good to include representative target NCBI accession numbers in description (i.e., what species do these primers bind).

**Yes, this was Lotmaria passim.**

20. Line 111-112 Methods
Was RNA isolated from all 25 mL or an aliquot of the total sample? If not, what was the volume of honey bee homogenate used for RNA isolation? What was RNA concentration after RNA isolation and DNase I treatment, since relative abundance calculations are dependent upon standard RNA amounts used for cDNA synthesis (i.e., as opposed to a volume containing an unknown amount of RNA. Unless all qPCR is relative, or based on RNA amounts in RT it is difficult to estimate virus copy number. The same information is required for individual bee samples, or at least the amount of RNA used for RT for each bee sample.

***We have clarified the extraction method, showing that while 25 ml of lysis buffer was needed to effectively blend the 50 bees, only a small fraction of this lysate was used for the resulting phenol-chloroform extraction. As in many/most surveys that part of the study does not purport to show absolute viral copy numbers, but instead dCT, the relative amount compared to an endogenous honey bee standard. This metric, when primer efficiencies for both the host and pathogens are the same, can accurately present differences in disease loads across samples.**

21. Lines 148+. The virus inoculum should be characterized. What viruses (or virus RNAs) were detected in the inoculum preparation? RNA should be extracted from a virus aliquot and relative virus RNA amounts should be quantified. It would be best to sequence the virus inoculum.

***We have quantified the inocula using the pathogens inferred from the screening but agree that a widespread metagenomic analysis will reveal additional microbes, as it tends to do. Our primary motivation for the inoculum presentations was to recapitulate adult bee mortality and to test whether each morbid bee carried an equally potent holobiont of viruses. We found heavy variance across inocula and at least an inference that a paralytic virus might covary with extreme virulence seen in inoculum CV5, we are hoping to explore these more fully and do not mean to discount the importance, not yet done in other inoculum-based trials to our knowledge, of more fully sequence-based workups.**

22. How did the authors rule-out pre-existing infections in pupae in their infection studies?
***The bees were all sourced from the sample colonies and if there were important latent viruses or other pathogens we would have seen the effects of these in both the heat-inactivated and PBS controls.**

23. The description of the association of IAPV with colony losses, could be improved based on current information and further consideration of that study, which compared samples from colony deaths to older/historic samples from other operations, and therefore did not identify accurately identify pathogens associated with CCD-affected colonies (e.g., Lines 413+“Israeli acute paralysis virus, was linked with colony losses in early samples from this loss episode16”; and Lines 57-58). It would be better to highlight findings on IAPV pathogenicity, (e.g., Chen et al PLOS Path 2014) and/or Cornman et al 2012 PLOS One).

*Those are indeed worthy references and it was not our intent to discount IAPV as a pathogen, just to say that with similar methods it was elusive.

Reviewer #2: The infectivity experiments are not sufficiently well-designed on their own, and they would probably be better framed in a different way. Right now, it reads as though they were meant to test if i.solates from symptomatic bees cause greater infection than isolates from asymptomatic bees when injected into pupae (a hypothesis that can’t be adequately tested with only 4 isolates). Moreover, the fact that the effect sizes in the infectious vs. heat-inactivated comparisons (Table 2B; which are presumably time-point matched) are so much lower than your comparisons of post-infection vs. time zero virus levels tells me that the injected pupae likely had fairly high levels of these viruses already. Background virus testing is not reported, so presumably it was not conducted. Because of these combined issues, I think these tests would be better framed in one or two lines as a preliminary confirmation that the method of generating inocula yielded infectious substances (i.e. confirming the viruses weren’t inactivated somehow during extraction).
**We are not sure we follow this fully, the [pupal injections were used as a surrogate to see which viruses were infective from the inocula, while the longevity trials were carried out with adult worker bees. For the latter, the adult bees treated with heat-inactivated isolates of the SAME inocula are truly key here, this demonstrates that a viable virus was needed to explain the cup mortality.**

Another question I have is the discrepancy between the methods and the results regarding the number of inocula generated from individual bees (methods say 12, results say 4 – which is it?). If 12, what happened to the others? Why were they excluded?
***yes while we collected 12 inocula it is correct that only 4 were used and are relevant.**
However, my biggest concern is that the adult survivorship assays were not designed in a way that is sufficient to point to the focal viruses as the cause of the broader U.S. colony losses in question. To achieve this, one would need to show that isolates from diverse sources derived from collapsing colonies cause significant mortality in individuals from a diverse range of colony backgrounds. Currently only four isolates (i.e. extracts from just four individual bees) have been tested on recipient individuals derived from a single colony, which is not sufficient to generalize the results. What if the bees from that colony had particularly poor immunity, or already had high background virus levels?
***This is a good point, but a counter is that bees collected from an entirely different part of the country and presumably not sharing the same environmental (pesticide) or genetic proclivities had exactly the same morbidity, in this case followed by death as did the bees sourced from California that were used to produce inocula. We have modified the discussion to make clear these four isolates were not representative of the entire country, but we do feel that the mortality they induced, and many-fold differences in mortality risk across even a small number of isolates, are both telling. We think if the injected bees had high background levels of viruse that we would have seen high mortality in, say, the heat-inactivated controls.**

Finally, the section on amitraz resistance is interesting, but only relevant if it led to higher Varroa levels in the collapsing colonies. Did it? Presumably, these colonies MUST have been sampled to determine Varroa infestation intensity, but these data appear to be missing from the manuscript.

***Mite counts are indeed recommended to beekeepers and can be insightful and we thank this reviewer and reviewer 1 for this. As above, we are not sure that mite counts in these colonies of various states of decline would have been accurate, it is very likely collapsing colonies would show higher mites in part because of dropping woprker numbers. Practically, for these triage samples the focus was on biological clues from the bees in colonies and in bees handpicked on the basis of symptoms, and we did not have concurrent or past Varroa loads. We do feel the trend toward varroa-vectored viruses as opposed to many other pathogens is important, and we feel that resistance is important even in operations where the beekeeper keeps mites down by 5+ treatments of Varroa, since this is both a huge expense and arguably harmful to bees. A management survey from this event with far wider reach than these samples or any sampling can provide supports the idea that beekeepers even when aware of mite levels, are conducting many treatments annually to attempt to control these mites (Nearman et al, 2025).**

Reviewer #3: The main conclusion is that high levels of three viruses likely contributed to the high mortality of colonies in the operations studied. However, the authors were (understandably) unable to provide pre-death samples. However, they could sample unaffected operations or compare their viral loads and prevalence with data from apparently healthy colonies from other surveys or experiments, or other jurisdictions. They do attempt to provide evidence for their conclusions from infectivity studies, which does help in this regard. While I suspect the authors are correct in that the high virus levels did play a role, the study is weakened by the lack of comparator levels, as well as the fact that they did not rule out other factors such as pesticides or nutrition. In the end, they provide posit that the operations that were affected by high colony mortality had high viruses (although no comparator data are discussed to substantiate this), but this does not demonstrate causation. In fact, their own data from the surviving colonies, which also had high viral loads, could be interpreted as evidence that the viruses did not play a role in the mortality. I strongly suggest that they authors incorporate some form of “control” viral levels, even if all they can provide is evidence from other regions, or historical titres. There must be some quantification of what is ‘normal’ to make the claim that the levels of DWV and ABPV are ‘unusually high’.

***Thank you. While we do not pretend to conduct an exhaustive literature review, we do a better job now of summarizing past evidence of viral levels in honey bee colonies to support our claim that these were unusual. The National Honey bee Health survey is arguably the best benchmark as it surveys across 42-47 states and has been in pace for 12+ years. The levels of ABPV in. this survey are generally quite low. We also cite three other ‘event’ (i.e., collection in times of colony loss) surveys that are informative and all show low levels for viruses in the Dicistroviridae, including ABPV, which have similar life histories and impacts..**

Reviewer #4: 1) The distinction and way in which DWV-A & B is reported needs to be consistent and improved. For example, line 239 notes that the “prevalent virus in these samples was Deformed wing virus (78%)” yet Table 1 shows that this is for variant DWV-A. It should be noted the relative absence of DWV-B (16.81%) in this study. Is this expected? Based on previous studies, including the 2024 paper (Hesketh-Best, P.J., Mckeown, D.A., Christmon, K. et al. Dominance of recombinant DWV genomes with changing viral landscapes as revealed in national US honey bee and varroa mite survey. Commun Biol 7, 1623 (2024). <https://doi.org/10.1038/s42003-024-07333-9>) that focused on the 2021 US survey that DWV-B (including the recombinants) where more prevalent. It should also be discussed the bias regarding the primer designs and how this relates to the DWV recombinants. Moreover, the levels of DWV-A appear to be greater than DWV-B (Figure 1)

***The Hesketh-Best paper is a great example of using more broad surveys to explore the bee holobiont, the most complete arguably to date, and we do not discount that approach. We, too, have marked fluctuations in DWV-A and DWV-B, here and prior, a main message of Hesketh/Best. The low levels of DWV-B in pooled samples of bees were indeed surprising to us (e.g., compared to Ryabov et al, from our group, using the original name of VDV-1 for what is now known as DWV-B). We would be more convinced this was due to primer mismatching if not for the fact that the same primers and methods were used on individual bees with and without illness, in the current study. In that case, the DWV-B levels were quite high and predictive. To us, this reflects a sampling issue that is a red flag for similar surveys in that pooled bee samples need not always reflect the most important agents, and that this bias can be present even when the lab diagnostics are exquisite (we do not claim ours were exquisite but they were similar to published works). We are just now grappling with that in our own efforts to address losses and do not have great answers other than, when time allows, the collection of morbid bees. This is a widespread challenge. Some studies, especially longitudinal ones but also including at-the-moment assays of hive strength, have found correlative differences in virus loads while others, including this one, have failed to do so.**

2) Figure 1 also indicates that LSV loads were greater than that of ABPV. As presented, the reader is led to infer that the primers used can detect all the variants of LSV. This is not true, right? And its impossible to assess (based on this data) what percentage of variants that are not being amplified or being missed by the primers used.

***These published primers are broad, and duly cited, but yes LSV as a species is impossibly broad to categorize by qPCR, one can only target the most prevalent lineages and use primers consistently study to study.**

3) Figure 2 and related tables are not being discussed as it relates to the data in Table 1 & Figure 1. Its notable that both DWV-A & B are associated with morbid bees from these surviving colonies (strong or weak… distinction not made). But considering that 78% of the colonies where DWV-A positive and 73% DWV-B negative, does this not tell us something about the role of DWV-A over -B in the overall role in colony health? Also, please include information if DWV-B (17%) co-occurred with the DWV-A (78%) colonies.

***DWV-A and B certainly co-occurred. Our best way to show that was via the clustering chart as like all surveys simply all combinations occurred, some bees high-A, low-B, some high both, etc. The raw data can certainly be perused for covariances and what we have in the past called ‘webs’ of pathogens and this is a very good point. We would be flattered if someone saw fit to make sense of those covariances. For this analysis we felt that the patterns for individual bees (which are the best for discovering a real biological cause of covariance) made as much sense as single-point metrics as they might have as chords of different species, but it is a lovely topic.**

4) As per point #3, Figure 3 is being interpreted that DWV-A & B co-occurred equally across all the morbid & healthy bees found across all the colonies. This was not so, correct? DWV-B was only in 17% of the surviving colonies. These individual bees only came from these 17% colonies. Is this correct?

***Again the pooled samples need not reflect the DWV A or B load of individual bees (each bee makes up 1/50^th^ of a typical pool and if most bees are uninfected at a given moment in time infected bees are easily missed), hence the value in doing the individual analysis and the perils, perhaps of just relying on pools. We were ourselves surprised by the contrast at a single point in time, but the techniques used were identical. We stand by each set of numbers and now feel they are not in conflict, just measuring a nested average (pools) versus a finer scale bee-level (sick versus healthy) look.**

5) Section “Infectivity and Induced mortality from viral inocula”: please don’t use the term “isolates” when speaking about inocula. Isolates should be used only when referring to individual specimens. In addition, only CV5 (wih the ABPV dominant virus) is described (lines 323-332). The CV3 and CV4 inocula is not described. Line 337 “Figure 3” needs to be “Figure 4”. The mortality curves for these two inocula are not dose dependent and the mortality is far less than CV5.

**This is a good point regarding isolates and we have corrected this. Inocula CV3 and CV4 are described with the rest in Table S3.**
6) The Amitraz resistance observations… these are based on only 39 individual mites from 18 colonies. Is the count low because mites were absent? Line 354, “Figure 4” should be “Figure 5”.

***We do feel this small set across 18 colonies is wide enough to spot-check resistance (as now made more clear in the text samples were spread across several operations that had come from multiple states).**

**Part III – Minor Issues: Editorial and Data Presentation Modifications**
Please use this section for editorial suggestions as well as relatively minor modifications of existing data that would enhance clarity.

***Thank you for all of these comments below and the immense time reviewers took to identify many places needing clarification and rewording. We have taken this list through the entire manuscript and it has made it truly better. In a couple rare cases we have kept our wording but only rarely. These changes , where not referenced specifically below, are noted in the tracked document.**

Reviewer #1: 1. Line 238 – Rephrase this sentence, which read as if 532 different pathogens were detected; suggest
“Samples from 113 colonies were tested for 11 potential pathogens (i.e., 1,243 total tests). There were 532 positive test results . .. . [[describe results in Table 1 more]] (Table 1).
**yes this was poor wording and is fixed.**
2. Lines 25-26 – maybe rephrase text

3. Line 34 check use of “$” or spacing

4. Line 38 – are other parasitic mites still detected in the US an/or in this sample cohort? If not, consider deleting that clause.

5. Gut parasites – should be updated / more specific for Plos Pathogens (i.e., Nosema ceranae, bacteria . …)

6. Maybe rephrase insecticides are a severe threat.

7. Suggest lower case for all virus names that do not include proper nouns, since italicized names indicate that the specific strain/species has been identified.
See website on how to write virus names - ICTV virus nomenclature
https://talk.ictvonline.org/information/w/faq/386/how-to-write-virus-species-and-other-taxa-names
There are a couple options for writing virus names (e.g., Deformed wing virus-A or deformed wing virus A), using capital letters for everything in the virus name.

8. Line 74 suggest changing “had been lost” to “died” to improve clarity for non-bee experts

9. Figure 3 – the A. and B. labels are missing from this figure.

10. Line 83 suggested changing “were indicated” to “were detected”

11. Consider changing the title to, “Viruses and vectors associated with honey bee colony losses”
Maybe I am too literal, but “tied to” did not seem accurate.

We feel they are indeed tied to these losses

12. In some test/tables “sacbrood virus” mistakenly has a space (“sac brood”).

13. Discussion Lines 425-426 “Coupled with our infection bioassays, we are confident that these results point to a causal factor for a large fraction of honey bee colony losses.” could be revised given that the inoculum used was not completely characterized (i.e., sequenced, tested exhaustively for other pathogens, etc.), but the sentence in the next paragraph addresses this.

14. Discussion Lines 429-430 “sequencing resources for these bees to scout for known and novel parasites and pathogens” suggesting revising to “sequencing libraries (or strategies) to test for additional pathogens and potentially identify new pathogens”.

15. Check placement of $ sign in text

Reviewer #2: Introduction:

Line 34: The $387 billion figure seems way too high. Are you sure this is not the figure for all animal pollination, not just honey bees?
**yes, for agricultural pollination generally, this has been cleared up.**
Line 41: I don’t know if it’s fair to say that the honey bees have evolved resilience, so much as beekeepers have selected for those production traits. Perhaps this should be reworded?
**We feel in this case it is a bee trait, arguably more from artificial than natural selection, but a phenomenon of bee evolution.**

Methods:
SI units for time are often not followed (e.g., "hours" when it should be "h"). Other small things like that that will be caught in copyediting, I'm sure

Line 132: Please list all pathogens that were tested here. In addition, were non-virus pathogens considered (e.g. Nosema)? This is clear upon reading the results, but it should be first listed in the methods.

Line 144: Please describe the inoculum/specimen origins in more detail. How many inocula did you produce and test? Where did the source colonies originate from (a map might help if it was across the US)? Was each inocula from a different colony? Reading on to the next paragraph, it seems that the answer to the first question is 12 inocula, but this and other details should be stated in the initial description.

Line 151 and elsewhere: Remember to replace “u” with “µ”

Line 170: Please list the concentrations used.

Line 180: Please revise this section to more clearly describe the sample sizes in each type of analysis, what are the dependent variables in each case, and the predictor variables utilized (including whether it was continuous or categorical, and if categorical, how many levels does it have and what were they).

Done

Line 181: R versions and packages should be accompanied by the appropriate citations.

Line 183: How did you determine this? I ask because it is a common misconception that the data itself must be normal; rather, it is the residual data post-modelling. This is worth checking because if your residual data is in fact normal, redoing the analysis with parametric tests would give you more power to detect differences that were previously missed. The residuals indeed did not meet the conditions of normality.

Line 185: What is a “descriptive state” and how was it used in the analysis? This isn’t clear to me

Line 187: A PERMANOVA does not “measure group means,” per se. Perhaps reword.

Line 200: The random factor of “specimen” is probably best modelled as being nested within colony, in this case.

Results:

Line 239: Please specify whether this is A or B.

Table 1: Can you provide some justification for why you zero in on ABPV, DWV-A, and DWV-B as “focal pathogens,” when there are apparently other viruses with much higher prevalence than DWV-A? Also, where did these 113 colonies originate from (maybe a map would help)? Furthermore, in the methods, extraction of RNA was described, but not DNA, which presumably must have been done if Nosema and Trypanosomes are included in the pathogen panel. The methods should be updated to reflect this. Note also that “Nosema ceranae” has been misspelled. Finally, please define your limit of detection (i.e. what level qualifies as detected and non-detected).
*The term ‘focal’ is dropped since we in fact were quantifying all targets and while we collect those three together it is largely from the emergent data (individual bees for all three, pooled bees for DWV-A and ABPV) and the known linkages between DWV-A and DWV-B. With the addition of three more targets and the added incidences of AmFV using a more permissive primer we do feel we have given attention to all of the major candidates. All pathogens and parasites were screened with RNA, including those with DNA genomes, targeting expressed genes of AmFV, *Nosema* and *Lotmaria*. Detection was based on crossing fluorescence threshold prior to 50 cycles and showing the expected melt curve temperature.

Figure 1: Why are relative quantification data shown (relative to beta actin) when absolute quantification was conducted, as described in the methods? Also, it looks like some dots are being displayed twice (once as the unjittered “outlier” data points in blue and once as jittered data points in other colors). It would help to include another figure panel showing the data separated by operation, for the reader to be able to assess the variance across operations (even if there is no significant difference). And how many operations are we talking about? Finally, it is very difficult to parse out the colony strength groups by colored dots … plotting them each with their own boxplot next to each other would be a more effective way to show the data. Same goes for Figure 2.

* **Thatnk you for the suggested figure, which we have added to the Supplemental files. We used Delta – ct for quantification relative to actin to describe pathogen levels in pooled and individual bees, a standard for bee pathology screens. We only calculated absolute quantification for** **the four viral inoculums, as describing dosage as deltaCT would not communicate well. We had reasons for that decision. We feel both measures, especially in the context of the massive number of studies of bee viral loads, offer a useful context for infection levels and (in the inocula) infectivity. For the inocula, we wanted to provide readers with some insight into the potential infectivity of these morbid bees as carriers. Also, arguably, for the inocula (which were treated far differently as they were ultra-filtered to remove bee cells and potential toxins) honey bee actin is a precarious metric for normalization.**


Line 260: It would be helpful to restate the test used in each case in the results text and/or figure legends. Also, some more punctuation may improve the readability here. It is not mentioned, but presumably a Bonferroni correction was used here too? Also, unless I missed it, somewhere in the methods it would be help to describe exactly how “morbid” and “healthy” were defined.

**Lines 144-146 connects described symptomatic bees with ‘morbid’ since that was the collective pathology observed in the field, i.e., they did not have a morphological pathology, just behavioral symptoms. Still, thereafter we tried to replace the word morbid following symptomatic to clear any confusion. “Healthy” bees were asymptomatic (not showing any clear morbidities).**

Line 261: What is a “symptomatic control” – do you mean “asymptomatic”? And how does being detected in 100% of symptomatic adults square with having only been detected in 19 out of 113 colonies?

***Yes, asymptomatic, thank you. We infer that sampling 50 nest bees missed infected nestmates, e.g., colony-level samples should be even larger to be effective/sensitive.**

Line 276: This sentence doesn’t make sense to me. It seems like words and/or punctuation are missing

Figure 3: This figure appears to be a screenshot. Please generate a new figure with appropriate resolution as well as a legend that describes the color gradient. The colors are confusing to me, as it seems to mean different things in different places. Also, please justify why only 7 of the 11 pathogens are shown here. For an unsupervised clustering analysis, one would expect all response variables to be included.
***Thank you, current submitted output is higher resolution. These targets had enough positive hits to be included across each colony**
Table 2: The data underlying these analyses would be best presented as a main figure, rather than a supplemental figure. Also, which values are for the 36 h time point and which are for 60 h?

Line 299: The way it’s written, I’m not sure if you’re referring to the inoculum or the injected pupa.

Figure 3: A PBS injection was described in the methods but not shown in the results. Please make these data explicit.

Line 332: This is a very big claim and I think it’s a bit misleading to frame the data in this way (as the contents of one bee will never be directly injected into 66 million bees in nature). Such statements should be made very carefully. In a lab setting, with additional stressors and using the most damaging infection method (injection), it’s not necessarily unusual for virus copy numbers in only the double or triple digits to kill adult bees when injected (IAPV is another common highly virulent agent). Using newly emerged bees in your experiments (i.e. before they have acquired their normal gut microbiome and subsequent immune activation) would also lead to higher than normal apparent virulence.
**We phrased it this way to show how extremely virulent that inoculum was on a standardized test, but have rephrased to be less dramatic.**

Discussion:

Line 418: Speaking of longitudinal studies, is there anything you can learn by putting your data in the context of the longitudinal data from the annual US national honey bee disease surveys?

**We have addressed this great point above and do now give it more context.**

Reviewer #3: Specific Comments:
Introduction:
• Line 53 It is nitpicky, but I feel this manuscript will potentially receive much attention, so I wish to help clarify / improve it as much as possible – I suggest considering a different word than ‘venerable” (deserving respect because of age, high position, or religious or historical importance) as venerable has a positive connotation.
• Lines 61-62 Awkward phrasing. Consider something like “losses… have been especially associated with arrival of the invasive honey bee ectoparasitic mite Varroa destructor in a region.”
• Line 67 Is there a missing space before “often”?
• Line 68 Here and elsewhere, consider “high mortality” rather than losses. The colonies are dead, not lost.

***We replaced losses with mortality in several places, however feel as a commodity term losses is more standard, and for historical and present sudden decreases in worker bee numbers losses can cover both absconding (admittedly we fee this is rare) and mortality outside of the hive. Where known bodies are involved we use ‘mortality’.**
• Lines 68-76 Please provide more detail on how these losses were reported, and to whom, and describe the data. It is a basic assumption of the paper that there was a massive die-off, so this needs to be described in a more fulsome way rather than just referencing the Project Apis m website.

**There is now a citation beyond the community survey and that is referenced.**
• Line 62, 87 You can abbreviate Varroa to V. destructor after the first mention, or Varroa as in line 66. In fact, you have already said it is a widespread bee parasite, so that can be deleted as well.
• Line 91 Consider “compromised honey bee colony health” rather than “bee declines” which could pertain to populations or species.
• The introduction is well-written, and the authors have taken care in their wording. I appreciate that despite the significance of the mortality, they have not resorted to alarmist wording or unwarranted extrapolations as was seen with CCD.
• However, I would encourage the authors to broaden their perspective beyond the US. What was experienced this winter by beekeepers in other regions (e.g. Canada and Mexico in N. America), and how does this die-off compare in scope or timing to other previous ones globally (e.g. Bailey’s papers on Isle of Wight disease)?
• E.g <https://capabees.com/shared/CAPA-Statement-on-Colony-Losses-2023-2024_FV.pdf>

Materials and Methods
• Please describe the sample collections in more detail in the current submission, rather than relying only on the referenced paper. How many operations / colonies/ bees, from where, how were they selected etc. Please provide enough detail that a reader can understand the population from which your samples were drawn.
• Lines 96-96 This is not informative: “… all samples were collected individually when not collected as pooled samples”
• Line 99 Please describe how they were scored in more detail. What was the scoring system, what were they scored for, specifically?

We used a consistent meqasure from past colony triage event (e,g,m colony screening described in Lamas et al., 2024). **Full coverage of worker bees across > 10 frames of bees was strong. Dwindled or fewer than 6 frames of bees with low density was weak. Medium colonies had between 6 and ten frames of worker bees.**

• Line 100 How was this randomised?
• Line 101 “brood (larvae and pupae) were” rather than was?
• Line 102 What signs of morbidity?
• Line 103-104 How were the stores evaluated and recorded?
• Please provide more information on the field inspections and collections in this paper. You can somewhat abbreviate if details are available in the other paper, but a reader should be able to get the basic idea without simultaneously reading the other paper.
• I am not qualified to review the molecular methods, so I will leave that to other reviewers.
• Line 144, 146 Viral inocula were
• Line 155 Purple-eyed
• Line 158 Do you mean World Precision Instruments microinjector? What model?
• Throughout – please use the Greek letter mu rather than a u in units to indicate micro
• Line 159 Isn’t this one time series with three time points? Or have I misunderstood?
• Line 164 Viral inocula
• Did you test what viruses were in the inocula?
• Line 165 If the bees were from a single colony, shouldn’t apiary then also be singular? Or was it one colony per apiary (and if so, how many apiaries?)
• Is this what was randomised in line 167 – bees from a single colony per apiary?
• Line 152-178 Please specify what the 4 inocula were? Have you pooled individual bees into the 50 ul aliquots x 4, each of which was delivered at three concentrations? Please clarify.
• Line 209 Please add more detail on where the mites came from and how these particular mites were selected.
• Line 213 …here you have switched to mu…
• I appreciate the urgency to publish this specific paper in a timely manner, however the M&M felt as though it was written in a rush, and lacked detail.
Results
• Line 240 “thanks to” is somewhat colloquial or this context
• Line 239 DWV levels should also be mentioned
• Line 240-241 The fact that ABPV levels were high compared to previous data is significant, but should be in the discussion rather than results (and should receive a more thorough treatment there). The results here should mention what the levels were (or refer to a figure / table).
• Line 242 …the presence of viruses
• Line 243 “..the viral load did not differ” please provide statistics in the test here
• It would be nice if you had a comparison to unaffected operations.
• Line 239-243 Were there sig dif in viral prevalence among viruses, or locations / operations?
• Table 1. What do you mean by focal pathogens?
• Figure 1. The axis fonts are small, even reading at 125%. Consider black boxes to distinguish them more from the blue dots for weak colonies. The figure heading should describe any abbreviations (e.g. ABPV, TRYPS). Describe ‘surviving colonies’ more – e.g. surviving colonies from US beekeeping operations experiencing high winter colony mortality. Explain the dark blue dots (presumably outliers) as well as the line (median?) and whiskers…
• Line 251 Do you mean overall # of pathogens? Or for each specific pathogen?
• Line 253 Do you mean no difference among operations in the load of individual pathogens? For any pathogen? What about prevalence?
• Please explain these statistical results more fully, and clearly, and in the text. Please fully describe the figure.
• R2 (superscript)
• Line 256-257 Please describe this more in the methods.
• Line 261 asymptomatic controls?
• Figure 2 See comments about Figure 1. Describe what the asterisks mean. In the figure heading, isn’t DWV-A also sig?
• Line 272-275 What about operations 1,4,6?
• Line 270 Extra “by” before operation
• Line 276 Do you mean respectively? Either way, I don’t think it is needed here. Do you mean among operations?
• Line 281 Across morbid bees (and I think symptomatic is preferable to morbid) or between morbid and healthy bees?
• Figure 3 Heading Perhaps symptomatic rather than “morbid” behaviors. Again, font size should be increased and more detail provided in the heading.
• Line 289 Do you mention these isolates / inocula identifiers in the methods? And please use consistent terms in the text and table 2.
• Do you describe analysing the viral components of the inocula in the methods (apologies if I just missed this). I was looking in the methods for info about the 4 inocula.
• Table 2 Heading Inocula (plural), heat-inactivated (hyphen)
• Line 331 extra comma after that
• Figure 3 heading Inocula (plural), field-collected (hyphen). There is no A and B on the figure as referenced in the heading?
• Lines 348-349 This is M&M
• Line 351 Far less common (no d)
• Figure 4 is unnecessary

Discussion
• Lines 361-363 To make the case that levels in affected operations are high, you need to compare the prevalence/ levels to either unaffected operations or to other survey/published data. How (quantitatively) do the levels compare?
• Line 363 Make the case that it is unusually high.
• Line 365 Reference that they are transmitted by Varroa?
• Line 366 Which surveys (citation).
• Line 367 Also Bahreini, Rassol, et al. "Arising amitraz and pyrethroids resistance mutations in the ectoparasitic Varroa destructor mite in Canada." Scientific Reports 15.1 (2025): 1587. And Marsky, Ulrike, et al. "Amitraz Resistance in French Varroa Mite Populations—More Complex Than a Single-Nucleotide Polymorphism." Insects 15.6 (2024): 390. As well as other references.
• More detail on these operations and their management is required in the methods. For how long and how often and at what does have they used Amitraz?
• Line 375 Previously you did not have umlauts on naïve (lines 325, 337)
• Line 392 “Viral infected bees live shorter lives.” Requires citation.
• Line 393 “crashing” is overly colloquial
• Lines 396 (Has it been kept in check…?)
• 396-407 This seems like a rabbit hole into acaricides that distracts from the main point of the paper. Many beekeepers in other countries (even commercial operations) do not reply on amitraz, so I would argue that there are already alternatives. Suggest this bit is shortened.
• Line 408-415 I was looking for this in the intro – move up.
• Line 415 There is a comma that should be superscripted
• Line 434 I strongly encourage you to strengthen this case. While you cannot get pre-loss data, can you provide data from operations that were not affected, or at least use quantitative evidence from other recent studies and surveys? What levels of these focal viruses are typically found in healthy colonies at least? We need a contrast to provide evidence that these values are high.
• Line 438- Definitely – see French, Sarah K., et al. "Honey bee stressor networks are complex and dependent on crop and region." Current biology 34.9 (2024): 1893-1903.
• In general, the manuscript felt rushed. More detail is required in many places, and more attention to the details of the text, headings, and formatting is required in numerous places. The discussion could be improved by a more thorough treatment of the literature, especially non-USA literature.

Reviewer #4: Line 169: change “95C” to “95oC”
Line 215: insert space between “30” and “cycles/s”

PLOS authors have the option to publish the peer review history of their article ([what does this mean?](https://gcc02.safelinks.protection.outlook.com/?url=https%3A%2F%2Ftrack.editorialmanager.com%2FCL0%2Fhttps%3A%252F%252Fjournals.plos.org%252Fplospathogens%252Fs%252Feditorial-and-peer-review-process%2523loc-peer-review-history%2F1%2F010f01978120cb69-4ab2aaa3-ccb0-427b-b74a-01ae685d107c-000000%2FHcq4hVHb1N4K9dahggsfZ2Fp3P_Ar0UL29ma5DMP8kU%3D215&data=05%7C02%7Cjay.evans%40usda.gov%7C06db2a43956c4a156f2708ddae1a0937%7Ced5b36e701ee4ebc867ee03cfa0d4697%7C1%7C0%7C638858149317503318%7CUnknown%7CTWFpbGZsb3d8eyJFbXB0eU1hcGkiOnRydWUsIlYiOiIwLjAuMDAwMCIsIlAiOiJXaW4zMiIsIkFOIjoiTWFpbCIsIldUIjoyfQ%3D%3D%7C0%7C%7C%7C&sdata=zdvcbMVtdytUQegdH367WFd%2F8MNlHgUcAISdtSgPJso%3D&reserved=0)). If published, this will include your full peer review and any attached files.


If you choose “no”, your identity will remain anonymous but your review may still be made public.


**Do you want your identity to be public for this peer review?** For information about this choice, including consent withdrawal, please see our [Privacy Policy](https://gcc02.safelinks.protection.outlook.com/?url=https%3A%2F%2Ftrack.editorialmanager.com%2FCL0%2Fhttps%3A%252F%252Fwww.plos.org%252Fprivacy-policy%2F1%2F010f01978120cb69-4ab2aaa3-ccb0-427b-b74a-01ae685d107c-000000%2FZYg_TRuInoGYmtOhJoHCyNkVooOVSCy3AcJrmJDUSiU%3D215&data=05%7C02%7Cjay.evans%40usda.gov%7C06db2a43956c4a156f2708ddae1a0937%7Ced5b36e701ee4ebc867ee03cfa0d4697%7C1%7C0%7C638858149317523966%7CUnknown%7CTWFpbGZsb3d8eyJFbXB0eU1hcGkiOnRydWUsIlYiOiIwLjAuMDAwMCIsIlAiOiJXaW4zMiIsIkFOIjoiTWFpbCIsIldUIjoyfQ%3D%3D%7C0%7C%7C%7C&sdata=o3PV6FW7MSXl%2Fm7VN78mzj1PDjfzPTAEHBSXQ26kTuU%3D&reserved=0).

Reviewer #1: No

Reviewer #2: **Yes:** Leonard Foster

Reviewer #3: No

Reviewer #4: **Yes:** Declan Schroeder
