# Supplementary material for: Viruses and vectors tied to honey bee colony losses
Source: PLoS Pathog. 2026 Feb 23;22(2):e1013939. doi: 10.1371/journal.ppat.1013939 (PMC12948310; doi:10.1371/journal.ppat.1013939)
Supplement: Attachment — Submitted filename: ResponseToReviewerComments_ThirdSubsmissionJE122225_ZSL.docx [file ppat.1013939.s004.docx]

Additional Editor Comments :

While the reviewers acknowledge the authors' efforts to address the comments, this manuscript still requires additional work. Three reviewers request that the authors take further effort to address all of the comments regarding the abstract, methodologies and discussion. The next re-submission should include a version of the manuscript that only highlights the changes that were made using a straightforward identification scheme, as the track-changes copy submitted here was difficult to follow. It seems comments between the co-authors are left on the track-changes copy, which makes the rebuttal appear rushed and incomplete.

*Thanks to the Editor and these reviewers for their careful critiques and prompts to make this a far better manuscript. We remain convinced that the analyses are timely and important, and have updated the resources and the text accordingly. We hope that the manuscript will now be suitable for publication in *Plos Pathogens.* The message remains consistent, that viral dynamics are the most likely cause of severe declines to commercial beekeeping operations, and that this unique survey of six of the largest commercial beekeeping operations in the US gives the most clear image yet of biotic factors during a period of colony collapse. We hope the changes and many additions address the raised concerns.

Journal Requirements:

1) We have noticed that you have uploaded Supporting Information files, but you have not included a list of legends. Please add a full list of legends for your Supporting Information files after the references list.

*The list of legends was posted at the end of the Supplementary File in our previous submission. PlosPathogens Submission Guidelines does not state where to place it otherwise. Thanks to your direction, a list of legends for Supporting Information has been added to the end of the manuscript after references as directed.

2) Please check the labeling of the supplementary tables as two tables are labelled as Table S4.
*Table S4 is only listed once, but our caption placement could be mistaken for the title of S5. This was corrected by adjusting the spacing throughout the Supplementary file.

Note: If the reviewer comments include a recommendation to cite specific previously published works, please review and evaluate these publications to determine whether they are relevant and should be cited. There is no requirement to cite these works unless the editor has indicated otherwise.


Reviewers' Comments:

Reviewer's Responses to Questions

Part I - Summary
Please use this section to discuss strengths/weaknesses of study, novelty/significance, general execution and scholarship.

Reviewer #1: Editors,

The manuscript entitled, “Viruses and vectors tied to honey bee colony losses” by Lamas et. al has been improved the review process, but requires additional attention, revision, prior to publication in PLOS pathogens. The authors address most of the reviewers’ comments, but there are some that still need to be addressed.

Reviewer #2: (No Response)

Reviewer #3: Summarized in my first review of this manuscript.

Reviewer #4: I commend the authors for their detailed rebuttal and subsequent revisions made to the manuscript. I do however still feel that the revisions made don't fully reflect all the points covered in the response to reviewers. The Discussion section does not go far enough to address the limitations of the study. Responses given in the rebuttal should be included in the Discussion. For example, when understanding the merits of PCR vs metagenomics, it would be appropriate for the authors to acknowledge (in line 362) that the inocula or resultant infections might not have been the result of the wild-type viruses but their recombinants. This is also true when it comes to the PCR results from the sampled colonies (Table1). As per their owns words in the rebuttal: "Applying these techniques [e.g. Hesketh-Best et al... metagenomic study] to individual colony samples or apiaries, when feasible, and connecting with colony status, will be insightful." and "As above, we would love to do that and also, of course, encourage others to collect and compare in this way from similar events."

*The reviewer is correct, we enthusiastically endorsed metagenomics as one path for discovery, and expanded this enthusiasm in our revision. This approach is one way to inform real-time bee loss events and we have been involved in several efforts to do so, including papers in 2007 and 2012 (Cox-Foster et al, <https://www.science.org/doi/abs/10.1126/science.1146498>, Cornman et al., <https://journals.plos.org/plosone/article?id=10.1371/journal.pone.0043562>) and ongoing efforts. Prior studies, including ours and in the past year Hesketh-Best/Schroeder, have identified important viral strains and recombinants. These insights improved, and will continue to improve, the ‘target set’ for qPCR analyses, especially in view of recombinants and variants for the DWV clade. We welcome those discussions and have made clear in the current manuscript that diagnostics as a whole are fluid and evolving.

We still believe, for both pragmatic and statistical reasons, that qPCR assays are vital for analyzing the hundreds of independent samples needed for statistical rigor, at least within the budgets of most projects today. That reflects the state of the science, internationally, i.e., we have not yet found studies that connect RNASeq-based differences in pathogens to major loss events, without statistical confirmation by qPCR. The importance of DWV recombinants is not lost, and yet we feel that our differing results for DWV-A and DWV-B based on single-point qPCR assays are informative and consistent with the biologies of those viruses. This is arguably because we used established primers for DWV-B that target the capsid protein region and, as our lab and Dr. Schroeder’s lab have shown, there is an asymmetry whereby many of the most successful recombinants carry DWV-B capsid proteins and DWV-A enzymes. We have added a screen for an additional virus suggested by the reviewers (Apis mellifera filamentous virus) along with the parasite *Acarapis woodi,* and two bacterial pathogens. We feel that the screening of a total of ten viruses and three eukaryotic parasites reflects a robust panel for honey bee disease. We stand by the fact that using qPCR for these important species is informative in this case and is the current recognized standard.

Its perfectly understandable that time and limited resources led to the choice of approaches used, however, given the resolution of sequencing over PCR as a surveillance tool, especially with the discovery of recombinants in honey bees, this study needs to acknowledge these limitations.

*As above we feel that we have given a balanced discussion of the merits for these approaches and our efforts for a survey of this scale are at or exceeding current efforts to direct the search for mortality drivers. The use of qPCR allowed this to be a more nimble analyses of the problem, offer more direct contrasts with multiple prior studies, and in our view provide telling quantitative data.

Part II – Major Issues: Key Experiments Required for Acceptance
Please use this section to detail the key new experiments or modifications of existing experiments that should be absolutely required to validate study conclusions.

Generally, there should be no more than 3 such required experiments or major modifications for a "Major Revision" recommendation. If more than 3 experiments are necessary to validate the study conclusions, then you are encouraged to recommend "Reject".

Reviewer #1: Points to clarify or address before publication include:
1. The authors should revise abstract based on previous reviewers’ feedback (i.e., Reviewer #4).
Specifically, authors should either remove claims made in the abstract that symptomatic bees are the best to sample the cause of honey bee colony losses, or include some more discussion about this claim. The affected/symptomatic bees don’t make up a high percentage of the colony. How do the authors know that the viruses associated with these bees are the cause of colony losses? It may be that some rewording in the abstract is needed, and additional clarification in the discussion. This point was made by Reviewer #4, but the abstract was not updated in the re-submitted version.

*The abstract revision has now been done, thank you. We had revised the text with our arguments for using the analysis of individual bees, when possible, and feel this is a key strength of our work. While using pooled samples will continue to be a useful metric in many, and perhaps most, cases, we wished to share the insights from this study that indicate a search for symptomatic bees, while tedious, can provide insights that were less pronounced, or missed, in pooled samples. We were surprised by this result and indeed have spent years conducting pooled surveys, including the US National Honey Bee health Survey. Both strategies have merits, one (pooled bees) is clearly better as a colony-level single datum metric.. but we learned a substantial amount in this study, we feel, by doing both. Individual analyses of bees with *morphological* pathologies (deformed wings) have long showed the connection with high virus levels. A 2025 paper, that we now cite, has similarly concluded that this cause is not apparent at the colony level, presumably for the same sampling reasons. Our study is we believe the first to show different patterns in bees at the moment of colony declines, and in bees showing behavioral but not morphological signs of viral disease.

2. Did the authors indicate from which colonies the mites were obtained? This data would enable coupling of colony virus data with mite information, maybe as a Supplemental Table or Figure (rev Rev #1 point 3).

*Thank you, this was updated in the result sections. 18 colonies, approximately half which were deceased provided the mites analyzed here. The intent was to get a snap shot of mite resistance, not to provide correlates with specific losses. Indeed by finding 100% presence of the resistance genotype we feel that mites in these commercial beekeeping colonies have, in the past several years, have responded to the repeated amitraz treatments inherent to commercial beekeeping.

3. Did the authors see “a strong discrepancy between morbid and healthy bees based on qPCR” between DWV-A and DWV-B? (Reviewer 1 – point not addressed).

*We are sorry if this was not addressed fully in the rebuttal, but we did improve the language for this analysis. In fact we show a strong discrepancy between morbid and healthy bees, and this discrepancy is found for *both* DWV-A and DWV-B. The subtlety, as described in lines 302+ is “While DWV was generally linked to morbidity, and both DWV strains clustered together, some cohorts of morbid bees had high levels of both viruses, while others had high levels of only DWV-B”. We left that text in as it was intriguing, and fits a general observation that DWV-B, at times, has higher virulence, but we feel both viruses play into the observed risk of disease.

4. Characterization of inoculum still requires further clarification in text and/or supplemental table. It is not clear what virus tests were performed. The supplemental table should include all test and a “-“ for below detection levels.

*Thank you, we have addressed this comment by updating the supplementary with screened viruses, and the B-actin detections

5. Host RNA would pass through a 0.2 uM (200 nM) filter. Re-consider request of Review 1 to use host housekeeping gene.

*Thank you, we have addressed this comment by updating the supplementary with screened viruses, and the B-actin detections.

6. Further correct/clarify, it seems inaccurate to stat that (“1/15,000 of the original bee”), maybe
(“1/15,000 of the original bee lysate”), but not of a bee. The authors should just refer to the “dilutions of virus suspensions” or inoculum dilutions.

*This has been completed.

7. The authors should avoid the use of “bee equivalents” in the text, although now defined in methods section it is a strange way to refer to inocula, using the dilution factor would be better.
Have you ever read “human” or “mouse” equivalents for virus inoculum in other scientific studies?
We have changed this wording to the more standard ’10^-8^, etc. dilution from the original bee isolate throughout, as requested.

8. To better address Review 3’s comment 2, the authors should clearly state, which LSV variants are detected using the qPCR primers utilized in this study.

*The LSV primers were designed to amplify broadly based on RNASeq data for this virus (https://peerj.com/articles/9424/), and are capable of amplifying LSV2, LSV3, and LSV4 variants.

9. Line 168-169 methods were NOT updated as indicated in response to review. The authors need to include how much of the 25 mL plus 50 bee homogenized sample was used for RNA extraction

*We took a practical amount of fully mixed homogenate (620ul) for RNA extraction. The final product was normalized against the house keeping gene (B-Actin). We did not change the protocol from this cited reference, the Bee Book. We adjusted the in line text to be more detailed.

10. Excel with RNA data, and qPCR data should be included as supplemental data, as requested by Reviewer 1.

*done.
11. Line 276 (and all other instances). As described by Reviewer 1, “titers” is not accurate for the data presented in this study – and should be corrected throughout the manuscript.

*done.

12. Line 316 – virus names do not need to be capitalized - See reviewer 1’s comments on virus nomenclature (ICTV) and correct text, as needed.

*Done.

Reviewer #2: (No Response)

Reviewer #3: Summarized in my first review of this manuscript.

Reviewer #4: No major revisions required except for a Discussion section that covers the limitations of the study design.

Part III – Minor Issues: Editorial and Data Presentation Modifications
Please use this section for editorial suggestions as well as relatively minor modifications of existing data that would enhance clarity.

Reviewer #1: Minor points to clarify or address before publication include:
1. The quality of writing was improved, but could still benefit from editing by a professional scientific writer.
For example, Line 73 – “in the face of” .
In addition, the previous reviewer’s comments were not fully addressed in revised version
*We have updated the wording throughput, and believe we have addressed all of the reviewers’ comments more completely in this version.

2. Other corrections needed, but not limited to, include:
Line 114 – “Deformed wing virus” should not be capitalized

*We corrected this throughout the text.

Line 116 and throughout “Varroa” should be “V. destructor”
*We have used the full species name in the abstract. In the main text body we have first named the species in the Introduction and then stated ‘hereafter *Varroa*’, since the genus name is definitive for the US.

3. Primer tables should include a column with citations for each primer set.

*Done.

4. Table S4 and figures – still indicated “TRYPS” when it should be Lotmaria passim (update throughout)

*This has been corrected throughout the document.

5. Figure S2 – uses “titers” which in not accurate, it is relative RNA abundance. (See previous reviewers’ comments)

*This has been changed to abundance.

6. Supplemental dwindled colony, change “unites” to “combined colonies”

*This has been done.

7. Survivorship bias – still has “Figure xxx)”; the authors need to re-review the text and correct any additional mistakes, I did not take the time review it for mistakes.

*Thank you, this has been corrected throughout.

8. Line 229 remove “by hand”

*Done.
9. Lines 238-239 “A no-inject negative control was utilized (N = 16) to indicate if damage was incurred by the
shunt injection. incurred damage” , requires revision – maybe “A control group that was not-injected (n=16) served as a negative control for damage incurred due to injection in the mock-infected group (n=XX).”

*Done.

10. Line 254 (and throughout) – categories “Strong, Medium, and Weak” do not require capitalization.

*We prefer to keep these capitals in as it is not an adjective but a formal category, as defined.

11. Line 317 define “IQR” in text

*We added a description of how we calculated IQR along with description on line 217 in the Material and Methods section under the statistical analysis subsection.

12. Line 350 “The source of individual bees (beekeeper operation) did not have a statistically significant
effect on viral targets DWV-A or DWV-B” is an odd way to write this, consider “The prevalence and abundance of DWV-A or DWV-B in individual bees from different beekeeping operations” (or something similar).

*done.

13. The use of “viral target” should be revised throughout, it seems the virus name can be substituted in all (or most) instances.

*done.

14. Line 371 and/or methods and figure caption should better indicate what viruses the inoculum were tested for; this is NOT indicated in Supplemental Table S3.

*Thank you, we have addressed this comment by updating the supplementary with screened viruses, and the B-actin values as another metric alongside initial volumes from the sampled bees.

15. Line 474-475 - Suggest changing “Israeli acute paralysis virus, like close relatives Kashmir bee virus and Acute bee paralysis virus, remains closely monitored and is known to be highly virulent for honey bees bees” to either
“Israeli acute paralysis virus, which is phylogenetically associated with Kashmir bee virus and Acute bee paralysis virus, is often monitored due to its high virulence in honey bees”. Or Israeli acute paralysis virus, which is phylogenetically associated with Kashmir bee virus and Acute bee paralysis virus, is highly virulent and thus often monitored.
Or – do you want to indicate that all three are monitored? If so, reword.
*Yes we wanted to note that all three have received heavy scrutiny, the sentence has been changed to “Israeli acute paralysis virus and close relatives Kashmir bee virus and acute bee paralysis virus are known to be highly virulent for honey bees and, hence, are frequently screened in honey bee colonies”.
16. Line 476 should be revised “Here we show a preponderance of RNA viruses in bees collected in the midst of . ..” again is odd wording.

*New wording: Multiple known RNA viruses were quantified in bees collected in the midst of a colony collapse event that impacted a majority of US-managed honey bee populations.

17. Line 497 “. . . led to replicative viruses and high pathogenicity.” Should be revised, maybe “. resulted in productive infection and high pathogenicity.”

*Done.

18. Line 539 “Varroa destructor is considered the most impactful factor for honey bee colony survivorship. . “ consider revising to “Varroa destructor infestation levels above 3% negatively impact honey bee colony survival, and mites are one of the largest concerns for the beekeeping industry. High mite infestation coupled with RNA virus infections are particularly damaging.” – or some other revision.

*We simplified this sentence to: When coupled with damaging RNA viruses, *Varroa destructor* is the primary threat for managed honey bee colonies [16 ].

19. Line 547 add citations to the following “Virus-infected bees live shorter lives.”

*Done.

Reviewer #2: (No Response)

Reviewer #3: Summarized in my first review of this manuscript.

Reviewer #4: none

PLOS authors have the option to publish the peer review history of their article ([what does this mean?](https://track.editorialmanager.com/CL0/https:%2F%2Fjournals.plos.org%2Fplospathogens%2Fs%2Feditorial-and-peer-review-process%23loc-peer-review-history/1/010f019a4e8c2b9d-6f0aab5f-a08d-401c-8da4-ed354232c5d2-000000/EaBtMOqjL7lzTSovEjUJXHsxUECzeh4imodzIMjcgyg=235)). If published, this will include your full peer review and any attached files.

If you choose “no”, your identity will remain anonymous but your review may still be made public.

Do you want your identity to be public for this peer review? For information about this choice, including consent withdrawal, please see our [Privacy Policy](https://track.editorialmanager.com/CL0/https:%2F%2Fwww.plos.org%2Fprivacy-policy/1/010f019a4e8c2b9d-6f0aab5f-a08d-401c-8da4-ed354232c5d2-000000/qaN_yNAoKTgK5RbAIYcprguVTDP3uL7CtT9kCc1vD18=235).

Reviewer #1: No

Reviewer #2: Yes: Leonard Foster

Reviewer #3: No

Reviewer #4: Yes: Declan Schroeder

[NOTE: If reviewer comments were submitted as an attachment file, they will be attached to this email and accessible via the submission site. Please log into your account, locate the manuscript record, and check for the action link "View Attachments". If this link does not appear, there are no attachment files.]

Figure resubmission:

While revising your submission, we strongly recommend that you use PLOS’s NAAS tool (<https://ngplosjournals.pagemajik.ai/artanalysis>) to test your figure files. NAAS can convert your figure files to the TIFF file type and meet basic requirements (such as print size, resolution), or provide you with a report on issues that do not meet our requirements and that NAAS cannot fix.

After uploading your figures to PLOS’s NAAS tool - <https://ngplosjournals.pagemajik.ai/artanalysis>, NAAS will process the files provided and display the results in the "Uploaded Files" section of the page as the processing is complete. If the uploaded figures meet our requirements (or NAAS is able to fix the files to meet our requirements), the figure will be marked as "fixed" above. If NAAS is unable to fix the files, a red "failed" label will appear above. When NAAS has confirmed that the figure files meet our requirements, please download the file via the download option, and include these NAAS processed figure files when submitting your revised manuscript.

Reproducibility:

To enhance the reproducibility of your results, we recommend that authors of applicable studies deposit laboratory protocols in [protocols.io](http://protocols.io), where a protocol can be assigned its own identifier (DOI) such that it can be cited independently in the future. Additionally, PLOS ONE offers an option to publish peer-reviewed clinical study protocols. Read more information on sharing protocols at <https://plos.org/protocols?utm_medium=editorial-email&utm_source=authorletters&utm_campaign=protocols>
